# Time-Varying Effects of Meteorological Variables on Malaria Epidemiology in the Context of Interrupted Control Efforts in the Amazon Rainforest, 2000–2017

**DOI:** 10.3389/fmed.2021.721515

**Published:** 2021-09-29

**Authors:** Gabriel Carrasco-Escobar, Jazmin Qquellon, Diego Villa, Renato Cava, Alejandro Llanos-Cuentas, Tarik Benmarhnia

**Affiliations:** ^1^Health Innovation Laboratory, Institute of Tropical Medicine “Alexander von Humboldt”, Universidad Peruana Cayetano Heredia, Lima, Peru; ^2^Herbert Wertheim School of Public Health and Human Longevity Science, University of California, San Diego, La Jolla, CA, United States; ^3^Facultad de Salud Pública y Administración, Universidad Peruana Cayetano Heredia, Lima, Peru; ^4^Instituto de Medicina Tropical “Alexander von Humboldt”, Universidad Peruana Cayetano Heredia, Lima, Peru; ^5^Scripps Institution of Oceanography, University of California, San Diego, San Diego, CA, United States

**Keywords:** meteorological variables, malaria epidemiology, *Plasmodium vivax*, *Plasmodium falciparum*, interrupted malaria control intervention, Amazon rainforest

## Abstract

Successful malaria control interventions, mostly based on the training of health workers, distribution of insecticide-treated nets, and spraying, decrease malaria incidence; however, when these interventions are interrupted, a resurgence may occur. In the Peruvian Amazon, after discontinuing the control activities implemented by the PAMAFRO project (2006–2010)-a Global Fund-sponsored project for the strengthening of malaria control and surveillance in multiple countries in Latin America– malaria cases re-emerged dramatically. In parallel, meteorological factors determine the conditions suitable for the development, reproduction, and survival of mosquito vectors and parasites. This study hypothesized that interruption of malaria interventions may have modified the meteorological-malaria relationships over time (i.e., temporal changes in the dose-response between meteorological variables and malaria incidence). In this panel data analysis, we assessed the extent that relationships between meteorological variables and malaria changed temporally using data of monthly malaria incidence due to *Plasmodium vivax* or *P. falciparum* in Loreto, Peru (2000–2017). Generalized additive models were used to explore how the effects of meteorological variables changed in magnitude before, during, and after the PAMAFRO intervention. We found that once the PAMAFRO intervention had been interrupted, the estimated effects (dose-response) of meteorological variables on incidence rates decreased for both malaria parasite species. However, these fitted effect estimates did not reach their baseline levels (before the PAMAFRO period); variations of time-varying slopes between 0.45 and 2.07 times were observed after the PAMAFRO intervention. We also reported significant heterogeneity in the geographical distributions of malaria, parasite species, and meteorological variables. High malaria transmission occurred consistently in the northwestern provinces of Loreto Department. Since the end of the PAMAFRO period, a higher effect of precipitation and actual evapotranspiration was described on *P. falciparum* compared to *P. vivax*. The effect of temperature on malaria was greater over a shorter time (1-month lag or less), compared with precipitation and actual evapotranspiration (12-month lag). These findings demonstrate the importance of sustained malaria control efforts since interruption may enhance the links between meteorological factors and malaria. Our results also emphasize the importance of considering the time-varying effect of meteorological factors on malaria incidence to tailor control interventions, especially to better manage the current and future climate change crisis.

## Introduction

Malaria remains a relevant public health problem, despite being a preventable and treatable infectious disease. During 2019, 229 million malaria cases were reported worldwide and ~139 million people were at risk of acquiring malaria in Latin America, where more than 90% of cases occur in countries with Amazon rainforest, such as the Bolivarian Republic of Venezuela (53%), Brazil (20%), Colombia (10%), and Peru (5%) (1). In this area, most (75%) cases are caused by *Plasmodium vivax* species and transmitted by the primary malaria vector *Nyssorhynchus darlingi* (also known as *Anopheles darlingi*). In the Peruvian Amazon region, malaria is the most important vector-borne disease, with most cases (93.1%) located in Loreto Department (2). Although the annual incidence rates (AIR) in this area decreased from 59.18 per 1,000 inhabitants in 2005 to 11.59 in 2010, a sharp increase was observed in the subsequent years, reaching a peak of AIR of 42.69 in 2013 for both endemic species, *P. vivax* (AIR of 34.94) and *P. falciparum* (AIR of 7.75) (3), and with modest reduction in the following years. Social, economic, and weather variability were hypothesized as drivers of the rapid increase in malaria incidence in recent years (4, 5).

During the PAMAFRO project (2006–2011)—financed by external funding sources such as the World Bank and established in strategic Amazon regions such as Peru, Ecuador, Colombia, and Venezuela—malaria cases were successfully controlled. Training of community health workers for early diagnosis, monitoring, and treatment in first-level health facilities, use of long-lasting insecticide-treated nets (LLINs), and community education in prevention measures were critical to the success of the project and malaria control in these countries (6, 7). However, control interventions were neither widely distributed nor consistent over time in all districts of Loreto (3). The southern provinces of Loreto (Ramón Castilla, Requena, and Ucayali) benefited the least from this project. Even the district of Soplin (Requena Province), where the highest incidence of both malaria species was reported, did not implement community education. Simultaneously, training of health workers was only conducted during 2007–2008, the improvement of diagnosis only during 2007–2010, and the LLIN distribution during 2009–2010 (3). Regrettably, due to lack of sustainable funding, PAMAFRO strategies were discontinued in 2011, and the incidence of malaria increased markedly in all regions the following year (8, 9).

Studies have reported that intervention setbacks or interruptions affect the transmission and re-emergence of malaria (10). On Zanzibar island, Tanzania, after stopping indoor residual spraying (IRS) due to economic factors, malaria cases re-emerged drastically after a period with the lowest malaria burden (11). In the Mutasa district, Zimbabwe, reduced funding for IRS caused a resurgence of malaria (12). Political instability also disrupted control programs as demonstrated in a border area of Brazil where a change of government stopped malaria prevention activities among indigenous peoples, resulting in an outbreak during 2017 (13). Furthermore, in the context of climate change and variability, malaria incidence may be further influenced by meteorological determinants. Although previous studies evaluated the impact of meteorological factors and control interventions on malaria transmission (14, 15), none have assessed whether the dose-response effect of meteorological factors on malaria incidence varies over time (time-varying effect) and if the interruption of control intervention periods plays a role in exacerbating the trends of those time-varying effects.

Meteorological factors are essential components of the mosquito life cycle and parasite reproduction (16, 17). Mosquitoes need particular ranges of temperature (from 23° to 31°C) to transition successfully between each biological stage (18). Particularly, precipitation and humidity patterns are also crucial for adult and immature mosquito survival. For example, an increase in precipitation is often related to higher mortality in mosquito larvae (19, 20). However, rainfall also contributes to mosquito breeding sources (natural or artificial water pools), and even determines their spatial distribution (21). Lower evapotranspiration, defined as the flow of moisture from the soil that directly evaporates into the atmosphere and the water that vegetation transpires into the atmosphere, provides suitable habitat for mosquito larvae development (22). Ambient temperature is a regulator of the parasite biological cycle (23), i.e., high temperature can decrease the parasite extrinsic incubation period (EIP) inside the mosquito salivary glands and stomach (24). However, temperature affects *P. vivax* and *P. falciparum* differently (14). *P. vivax* can develop at a range of 15–30°C, showing less sensitivity to temperature changes compared to *P. falciparum*, which develops at a narrower range (18–30°C) (25). In addition, it has been hypothesized that temperature impacts *P. vivax* during the hypnozoite stage in the human liver until reactivation. The increase in temperature lengthens the latency time of temperate hypnozoite strains in the absence of other external factors such as fever due to other infections (26). In this context, documenting how dose-response relationships between meteorological factors and malaria incidence change temporally, especially in the context of intervention interruptions, is critical to provide updated epidemiological evidence to identify preventable outbreaks.

As malaria transmission is sensitive to meteorological conditions, the use of early warning systems (EWS) offers the opportunity to take proactive measures to reduce the impact of vector-borne diseases due to the forecasting of unusual temporal or spatial trends prior to the onset of an outbreak (27). For malaria forecasting, an EWS was constructed using temperature, humidity, and precipitation (28). This system identified an increase of malaria cases during warm and humid seasons compared to cold and dry ones. The lagged effect of temperature and rainfall also described the common seasonal patterns observed in malaria epidemics (29). However, endemic infectious disease areas are characterized by extrinsic time-varying factors (public interventions, social and political conflicts) that may affect the course of an outbreak, which would require regular updates of EWS parameters (30).

In this context, our study aims to assess the time-varying relationship between malaria and climatic variables and explore retrospectively whether deviations from the baseline trends were synchronic with the interruption of control strategies (such as the PAMAFRO project). We analyzed detailed data on *P. vivax* and *P. falciparum* malaria cases at a local scale between 2000 and 2017 in the Peruvian Amazon.

## Methods

### Study Design

We conducted a retrospective panel data analysis using secondary data of monthly malaria incidence in Loreto, Peru, and meteorological data derived from satellite imagery data to analyze the climate-malaria relationship over an 18-year period.

### Study Area and Population

Loreto Department, in northeastern Peru, covers 28.7% of the national territory. The political-administrative organization of Loreto is divided into eight provinces and 53 districts, with Maynas being the province with the largest territorial area and population density in the department, and Iquitos (the capital of Loreto), its most populous city. We included only 49 districts because four were created after the start of the data collection in 2000. According to the National Institute of Statistics and Informatics (INEI is the Spanish acronym) and the Peru National Household Survey (ENAHO in Spanish) in 2019, the total Loreto population was 883,510 inhabitants, of which 69.6% were urban dwellers. Overall, 32.2% of the population lives in poverty (7% in extreme poverty) and only 39.6% of households accessed basic public services (water, sanitation, electricity, and telephone) (31).

The health system in Loreto is composed of public and private clinics. In 2016, Loreto had 521 healthcare facilities (2.8% of the total national facilities), where each physician had to assist an estimated 1,086 inhabitants (32). Regarding health coverage, 85.4% of the population had health insurance, of which 66.6% were affiliated with the free integral health insurance (access to health establishment belonging to the Ministry of Health of Peru [MoH]) (33). For malaria, the diagnosis is carried out exclusively by MoH. However, health access is still deficient in indigenous and rural communities in the Peruvian Amazon, mainly due to transportation and monetary barriers (34, 35). Approximately 83.3% of the population had informal employment since the most common economic activities are based on agriculture, fishing, and mining (32). The geographical location of Loreto, in the Amazon basin, has a rainy tropical climate with high levels of humidity. The maximum temperature reaches 36°C (between December and March), and the minimum is 17°C (between June and July). The humidity and rain are constant throughout the year, with greater intensity between December and May (36).

### Data Sources

#### Malaria Passive Case Detection Data

Epidemiological surveillance of malaria in Peru, carried out by the General Directorate of Epidemiology of MoH, established mandatory weekly case reporting through NOTI software in all health facilities (37, 38). Most of the data were collected at health facilities (passive case detection). The data included malaria cases caused by *P. falciparum* and *P. vivax* species at the district- or province-level.

In Peru, the most common method for laboratory malaria diagnosis is microscopic examination of a thick and thin smear of capillary blood to identify *Plasmodium* presence and specific *Plasmodium* species, respectively. Thick and thin smear procedures (39) and treatment management (40) have been established in national guidelines provided by the Ministry of Health.

For this study, we collected monthly confirmed malaria cases classified by species during 2000–2017 in all districts of Loreto. Data from the four districts created since 2014—after data collection had begun—were included in the districts where they were formerly located. Monthly malaria incidence rates (MIR) were calculated as the ratio of malaria cases to the total population at risk of malaria.

#### PAMAFRO Study Description

The PAMAFRO project, sponsored by the Global Fund, focused on health worker training and community-based prevention measures and conducted four types of activities for malaria intervention throughout the Loreto region between 2006 and 2010 (41). First, improvement of malaria diagnosis, through training, monitoring, and constant evaluation of experienced and less experienced microscopists, purchase of new microscopes, and maintenance of existing microscopes (7), and implementing a diagnostic quality control system and active and passive search of cases. Second, the strengthening of malaria monitoring and treatment through the supply and availability of antimalarial drugs and inputs, treatment standardization, monitoring treatment adherence, evaluating drug efficacy, and training health workers (3). Third, community participation, through health promotion and education using graphic, radio, and audiovisual materials, formation of community groups to participate in health decision making, searching for community leaders to facilitate communication of behavioral change, and technical and financial support of community environmental management activities and financial support for prevention campaigns. Finally, the project integrated vector control by purchasing logistical support for the distribution of LLINs in all communities, identifying and eliminating mosquito breeding sites, and applying IRS (6).

Considering that the interventions were not consistently implemented over time, we defined dichotomous variables for each intervention type to identify whether it was conducted during each year in a particular district. Other control efforts carried out by national or international institutions during periods outside of the PAMAFRO project time frame were not considered for this study.

#### Meteorological Data

Meteorological data for terrestrial surfaces were obtained from the TerraClimate dataset (Climatology Lab of the University of California, Merced) based on climatic aided interpolations (at ~4-km spatial resolution) from 1958 to 2019 for several meteorological variables, which combine high-resolution spatial climatological normal from the WorldClim with time-varying data from Climate Research Unit version 4.0, and the Japanese 55-year Reanalysis (42). The meteorological variables used for the analysis were actual evapotranspiration (mm), precipitation (mm), runoff (mm), and maximum and minimum temperature (°C). The dataset was downloaded and processed using R software version 5.0.3 to produce monthly district aggregates for the meteorological variables by taking the mean cell values of the grid that pertain to each district in Loreto.

### Data Analysis

A descriptive analysis was conducted, where we first analyzed graphically the annual incidence rate variation of malaria by species between 2000 and 2017 at the provincial level, comparing the variation before, during, and after the PAMAFRO intervention. Data from the new province of Putumayo (2014–2017) was merged to their parental province, Maynas province. We also estimated the mean and standard deviation of the annual incidence rates by species and the annual values of the meteorological variables at the provincial level to compare their central tendency and dispersion throughout the study period. Finally, a repeated measures correlation analysis (43) was carried out over the observed values of the meteorological variables at a district level. For studies with repeated measurements of the same groups or individuals over time, the usual technique for a correlation analysis is to aggregate the observations for each group and calculate the correlation coefficient across the aggregated data. However, this may lead to spurious results if the inter-group associations differ from the intra-groups associations (Simpson's paradox). Repeated measures correlation analysis allows for isolation of the inter-district variability to better estimate the common within-district association of the meteorological variables.

Generalized additive models (GAM) were used to apply smooth functions over time to estimate the time-varying effects (i.e., monthly specific slopes) of the meteorological variables on the malaria case count at the district level. Separate models for *P. vivax* and *P. falciparum* were constructed using the same set of selected meteorological covariates to compare the results. The selected variables were the monthly actual evapotranspiration (mm), precipitation (mm), and minimum temperature (°C). Covariate selection for each meteorological exposure model was based on *a priori* knowledge of the structural dependence between these factors. Monthly district counts of new malaria cases were modeled using a Negative binomial (NB) distribution. This distribution was chosen primarily to address the presence of over-dispersion in the counts. Further discussion about the specific distribution can be found in the Supplementary Methods section. In addition, we built lagged versions of the models to analyze how the lagged effects of the meteorological variables changed over time. We lagged the values of the covariates by 1, 3, 6, and 12 months and built a different time-varying coefficients model for each lag value. We refer to the model without lagging as the base model. To make the models comparable (fitted over the same data set), we filtered out the first year (2000) since for the 12 month-lagged model we had observations starting at January 2001. Consequently, we fitted all the models with observations of 204 months, from January 2001 to December 2017. We then compared the base model with the lagged models using the Akaike information criterion (AIC) to determine which configuration best fit the data.

For each parasite species, the base NB additive model for the expected cases count was specified as follows:


$$\begin{array}{l} log\left[E\left(Y_{ij}\right)\right]=\alpha \;+{{\;\delta }_{0}\left(district\right)\;+\;\gamma }_{1}\left(t\right)\cdot aet_{ij}\;+\;\gamma_{2}\left(t\right)\cdot prcp_{ij}\\ \;+\;\gamma_{3}\left(t\right)\cdot mintemp_{ij}\;+log\left(pop2015_{i}\right), \end{array}$$

were *E*(·) is the expectation operator, and *Y*_*it*_ is the count of malaria cases for the *i*-th district (*i* = 1, ..., 49) in the *j*-th month (*j* = 1, ..., 204). The covariates *aet*_*ij*_, *prcp*_*ij*_, and *mintemp*_*ij*_ represent the values for the actual evapotranspiration, precipitation, and minimum temperature, respectively, for the *i*-th district in the *j*-th month. The parameter α is the intercept of the model. The variable *t* represents the continuous time over the period of study, and its values were generated by creating date-time values using the months and years of the measurements in the dataset and then converting them into numerical values. Further detail about the generation of these time values can be found in the Supplementary Methods section. The functions γ_1_(*t*), γ_2_(*t*), and γ_3_(*t*) are smooth functions over the time variable, and they act as time-varying regression coefficients for the covariates. These smooth terms in the model were approximated using *penalized regression splines*. These types of splines are penalized for their curvature complexity to prevent over-fitting. The degree of penalization is controlled by a regularization parameter, known in the GAM literature as the *smoothing parameter*, which can be estimated from the data along with the estimation of the whole model. The term δ_0_(*district*) is a random effect term for the districts to adjust for the repeated measures at this geographic level. Finally, the log-population of the districts in 2015 was added as an offset to the models to estimate incidence rates. We did not consider population density as an additional covariate of interest as we assumed no time changes in this variable during the study period. The configuration for the lagged models is the same, but using the lagged values of the covariates instead. The models were estimated using the implementation of the *restricted maximum likelihood* estimation algorithm done by Wood (44). The model fitting under all these specifications was done using the **mgcv** package of R software. Further details about the specifications of the GAMs are available in the Supplementary Methods section, but for a more comprehensive review of this topic, please refer to Wood et al. (45).

Before model fitting, we scaled the meteorological variables from 0 to 1, where 0 corresponds to the minimum observed value and 1 to the maximum. Scaling was done to obtain comparable estimates of the coefficients (i.e., standardize), and avoid convergence problems. Therefore, the value of a coefficient for a given meteorological variable at a specific point in time is the log-relative risk of the malaria incidence when the meteorological variable changes from its minimum value to its maximum.

The fitted values of the time-varying coefficients and their confidence intervals were plotted over time, highlighting the period of the PAMAFRO intervention. This strategy enabled a visual analysis of how the coefficients changed before, during, and after this period. For the base time-varying coefficients model, we built a table for the maximum and minimum fitted values and their confidence intervals on each period to facilitate analysis.

## Results

### Malaria and Meteorological Heterogeneities

**Figure 1:**
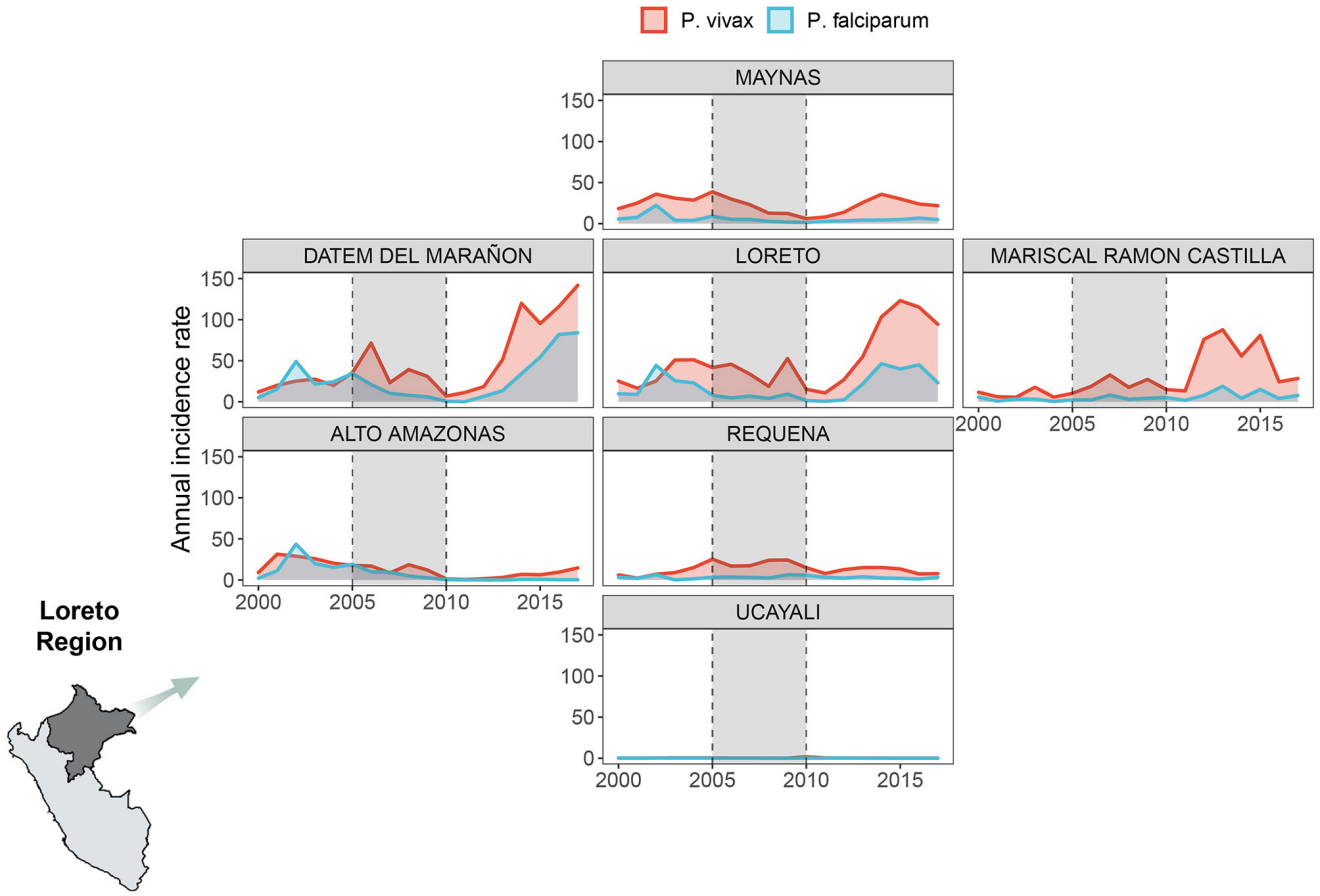
Annual malaria incidence rates variation by parasite species. Annual malaria incidence rates variation due to *P. vivax* (red) and *P. falciparum* (light blue) in 7 provinces of Loreto Region between 2000 and 2017. We created our own grid using the geofacet R package to represent the Loreto provinces: Maynas (first row), Datem del Marañon, Loreto, Mariscal Ramon Castilla (second row from left to right), Alto Amazonas, Requena (third row from left to right), Ucayali (fourth row). The period shaded in gray represents the PAMAFRO intervention.

Overall, 697,916 malaria cases were analyzed in all the Loreto districts between 2000 and 2017. Most of them (76%) were caused by *P. vivax*, while 24%, by *P. falciparum*. Over time, the AIR decreased between 2006 and 2011 for both malaria species exclusively during the PAMAFRO period. Figure 1 shows an important geographical heterogeneity of malaria epidemiology for both parasite species. At the sub-regional level, high malaria transmission occurred consistently in the northern provinces of Loreto (Loreto, Datem del Marañon, Mariscal Castilla, and Maynas) compared to the southern provinces (Requena, Alto Amazonas, and Ucayali). The highest mean AIR (per 10,000 population) of *P. vivax* malaria (Table 1) was in Loreto province (Mean-*M* = 50.3; Standard Deviation-*SD* = 35.5), whereas the lowest in Ucayali (*M* = 0.3; *SD* = 0.4). The highest mean AIR of *P. falciparum* malaria was in Datem del Marañon province (*M* = 26.2; *SD* = 25.9), the lowest in Ucayali (*M* = 0.03; *SD* = 0.02).

**Table 1:** Mean and standard deviation (SD) of the annual incidence rates by parasite species and the annual values of the meteorological variables in the 7 Loreto provinces between 2000 and 2017.

**Provinces**	**AIR**		**AIR**		**Actual**		**Precipitation**		**Runoff**		**Maximum**		**Minimum**	
	***P. vivax***		***P. falciparum***		**Evapotranspiration**				**Temperature**		**Temperature**		
	**Mean**	**SD**	**Mean**	**SD**	**Mean**	**SD**	**Mean**	**SD**	**Mean**	**SD**	**Mean**	**SD**	**Mean**	**SD**
Alto Amazonas	13.01	9.51	7.85	11.22	84.93	3.66	186.79	17.56	101.86	16.93	31.63	0.25	21.55	0.25
Datem del Marañon	48.16	42.14	26.17	25.86	86.14	4.28	197.68	18.52	111.55	17.85	30.37	0.34	21.11	0.32
Loreto	50.30	35.49	18.11	16.19	82.12	3.97	233.04	36.00	150.92	36.05	31.24	0.32	21.61	0.26
Mariscal Ramon Castilla	29.71	26.73	5.50	4.81	85.01	3.41	266.47	46.04	181.46	46.67	31.33	0.20	21.85	0.20
Maynas	23.39	9.74	5.64	4.60	80.13	3.52	275.59	67.55	195.45	67.50	30.87	0.29	21.61	0.21
Requena	13.44	6.65	3.16	1.72	82.41	4.15	210.67	31.10	128.26	30.11	31.87	0.22	21.38	0.22
Ucayali	0.26	0.37	0.03	0.02	85.80	3.37	160.20	17.50	74.41	16.62	32.62	0.25	20.74	0.25

*AIR, Annual incidence rates (per 10,000 population); Precipitation, actual evapotranspiration and runoff in millimeters (mm), maximum and minimum temperature in Celsius degrees (°C)*.

The variability in the distribution of meteorological factors between provinces during the period of study is presented in Table 1. Requena had, on average, the lowest annual mean value of actual evapotranspiration (*M* = 82.41 mm; *SD* = 4.15), while Datem del Marañon had the highest (*M* = 86.14 mm; *SD* = 4.28). Both provinces also had the greatest standard deviations of the annual mean values for this variable, meaning a broader variability. Ucayali showed, on average, the lower level of annual mean precipitation (*M* = 160.20 mm; *SD* = 17.50), whereas Maynas had the highest (*M* = 275.59 mm; *SD* = 67.55). Greater dispersion is shown in the annual values for this variable compared to the others, i.e., Maynas is the province with the highest dispersion. Regarding the annual mean of maximum temperature, on average, Datem del Marañon had the lowest level (*M* = 30.37°C; *SD* = 0.34) and Ucayali had the highest (*M* = 32.62°C; *SD* = 0.25). In the case of minimum temperature, lower levels of annual means were seen in Ucayali (*M* = 20.74°C; *SD* = 0.25) and the highest levels in Mariscal Ramon Castilla (*M* = 21.85°C; *SD* = 0.20). In addition, the outbreak of malaria cases following the interruption of the control efforts was accompanied by an increasing trend of the mean levels of the maximum and minimum temperature over most of the areas of study (Supplementary Figures 1–7). For instance, in Datem del Marañon, one of the provinces which had a mayor outbreak, the mean minimum temperature went from ranging from 20.30° to 21.57°C before the interruption to ranging from 20.7° to 22.06°C after the control ceased, and the mean maximum temperature went from ranging from 29.55° to 30.79°C before the interruption to ranging from 29.98° to 31.33°C.

Supplementary Table 1 shows the repeated measures correlation coefficients (*r*) and their 95% confidence intervals for the climate variables. The most noticeable finding is the almost perfect correlation between precipitation and runoff (*r* = 0.99; 95% CI: [0.99, 0.99]) within the districts during the period of study. Therefore, including both variables in a model for the malaria incidence rate would have caused multi-collinearity problems. Other variables that showed moderate to strong correlation were the actual evapotranspiration and maximum temperature (*r* = 0.56; 95% CI: [0.55, 0.57]), and maximum temperature and minimum temperature (*r* = 0.44; 95% CI: [0.43, 0.46]).

### Meteorological Time-Varying Effects

**Figure 2:**
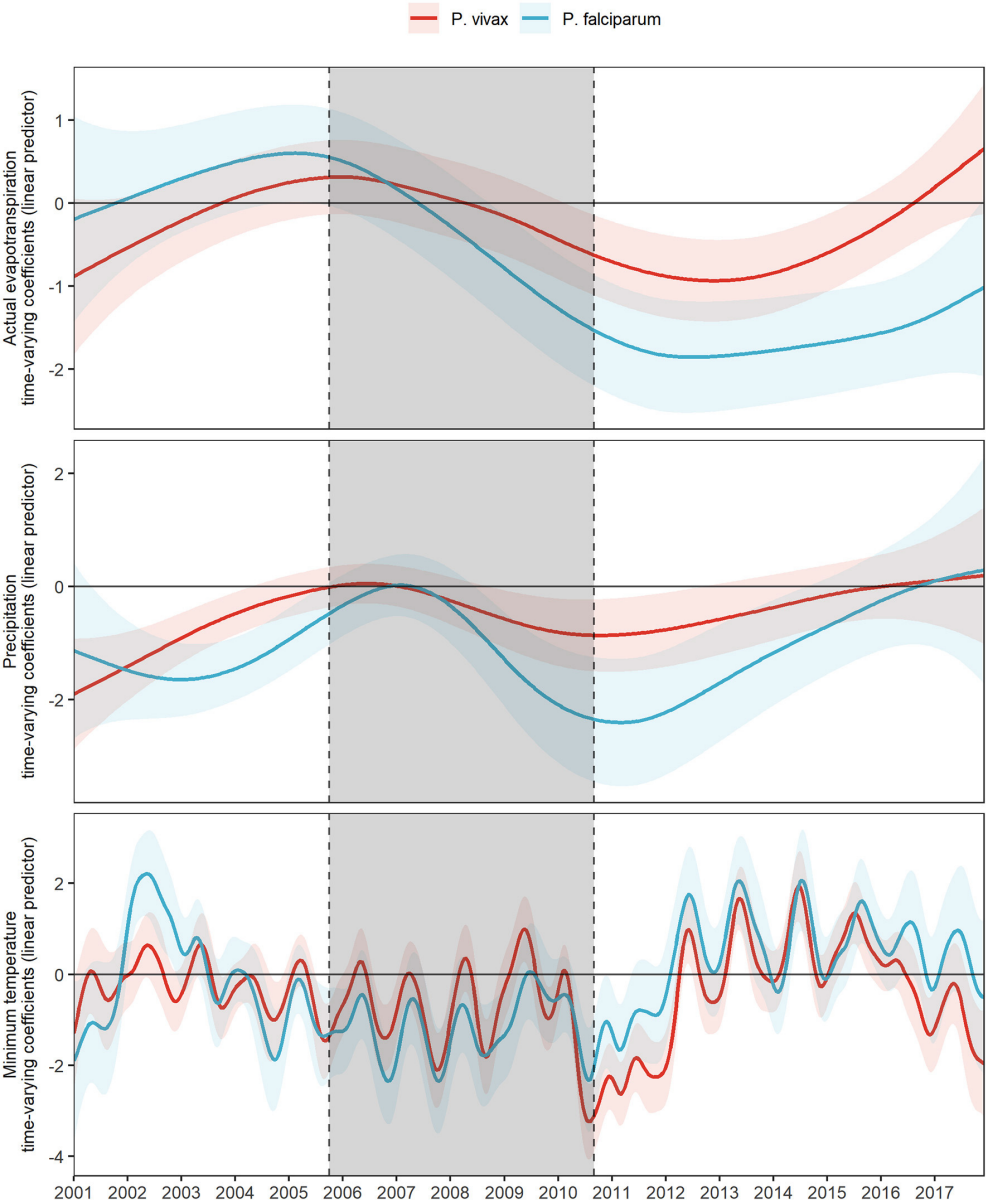
Fitted time-varying meteorological effects on MIR by parasite species. Fitted time-varying meteorological effects due to *P. vivax* (red) and *P. falciparum* (light blue) and their 95% CI (shading of each colored curve). The period shaded in gray represents the PAMAFRO intervention.

The fitted values of the time-varying coefficients for actual evapotranspiration, precipitation, and minimum temperature, along with their 95% confidence intervals, for both the base model for *P. vivax* and the base model for *P. falciparum* from 2001 to 2017 were plotted in Figure 2.

A concurrent exacerbation to a more negative effect of all meteorological variables on the log-relative risk occurs right before and right after the end of the PAMAFRO intervention (Figure 2). This exacerbation to a more negative effect seems greater for actual transpiration and precipitation for *P. falciparum* than for *P. vivax*, whereas the difference is less evident for the effects of minimum temperature. Table 2 presents the maximum and minimum values before, during, and after the PAMAFRO intervention of the fitted coefficients of the meteorological variables on each parasite-specific model. The effect of actual evapotranspiration on *P. vivax* began to have a progressive change from being not significant throughout the intervention to a more negative effect around mid-2010 (Figure 2 and Table 2). After the intervention, the minimum coefficient value was −0.94 (95% CI: [−1.43, −0.44]) at the end of 2012, and then transitioned to a non-significant effect between 2015 and 2016. On the other hand, the effect of actual evapotranspiration on *P. falciparum* transitioned from being not significant at the start of the intervention to a more negative effect around mid-2008, prior to the effect on *P. vivax*. It reached the value of −1.86 (95% CI: [−2.52, −1.19]) between 2012 and 2013, which is the minimum value throughout the whole period of study.

**Table 2:** Maximum and minimum fitted effects of the meteorological variables of the adjusted models for the parasite species within three periods: before, during and after the PAMAFRO intervention.

**Climate variable**	**Before**				**During**				**After**			
	**Min**		**Max**		**Min**		**Max**		**Min**		**Max**	
	**Estimate**	**95% CI**	**Estimate**	**95% CI**	**Estimate**	**95% CI**	**Estimate**	**95% CI**	**Estimate**	**95% CI**	**Estimate**	**95% CI**
***P. vivax***												
Actual evapotranspiration	−0.88	[−1.82, 0.06]	0.31	[−0.13, 0.76]	−0.62	[−1.11, −0.14]	0.31	[−0.13, 0.76]	−0.94	[−1.43, −0.44]	0.66	[−0.13, 1.44]
Precipitation	−1.90	[−2.88, −0.92]	−0.01	[−0.37, 0.34]	−0.87	[−1.50, −0.23]	0.05	[−0.30, 0.40]	−0.87	[−1.51, −0.23]	0.19	[−1.02, 1.40]
Minimum temperature	−1.46	[−2.29, −0.64]	0.66	[−0.03, 1.34]	−3.23	[−4.07, −2.40]	1.00	[0.28, 1.71]	−3.04	[−3.81, −2.26]	1.93	[1.17, 2.69]
***P. falciparum***												
Actual evapotranspiration	−0.19	[−1.43, 1.04]	0.60	[0.02, 1.18]	−1.53	[−2.20, −0.86]	0.55	[−0.04, 1.13]	−1.86	[−2.52, −1.19]	−1.01	[−2.08, 0.06]
Precipitation	−1.65	[−2.30, −1.00]	−0.48	[−1.03, 0.07]	−2.35	[−3.45, −1.25]	0.02	[−0.53, 0.57]	−2.41	[−3.53, −1.28]	0.29	[−1.72, 2.30]
Minimum temperature	−1.90	[−3.68, −0.12]	2.21	[1.28, 3.15]	−2.35	[−3.49, −1.20]	0.06	[−0.99, 1.11]	−1.88	[−2.98, −0.79]	2.07	[0.96, 3.17]

*95% CI: 95% Confidence Interval*.

*Precipitation and actual evapotranspiration in millimeters (mm), maximum and minimum temperature in Celsius degrees (°C)*.

A similar pattern was found for precipitation, the only difference being that its effect on *P. vivax* did not intensify much in magnitude at the end of the intervention, compared with its effect on *P. falciparum*. The latter increased significantly in magnitude toward a more negative effect, reaching the value of −2.41 (95% CI: [−3.53, −1.28]) at the first quarter of 2011, right after the end of the PAMAFRO intervention. Finally, it is apparent from Figure 2 that the effect of minimum temperature on the log-relative risk of malaria incidence presented a seasonal pattern with a stable level throughout the whole period of study. Two disruptions in the level of its effect are visible in the plot. The first one occurred between 2002 and 2003, where the effect of minimum temperature on *P. falciparum* had a substantial increase toward a more positive effect, and the second one between 2010 and 2012, where the effect on *P. vivax* increased toward a more negative effect. In the latter period, the effect of minimum temperature on *P. vivax* reached a value of −3.23 (95% CI: [−4.07, −2.40]) in 2010, the same year the intervention ended. Before 2010, the effect of minimum temperate on both parasite species tended to have a negative level, whereas, after 2012, it tended to have a positive level.

### Variation of Meteorological Time-Varying Lagged Effects

**Figure 3:**
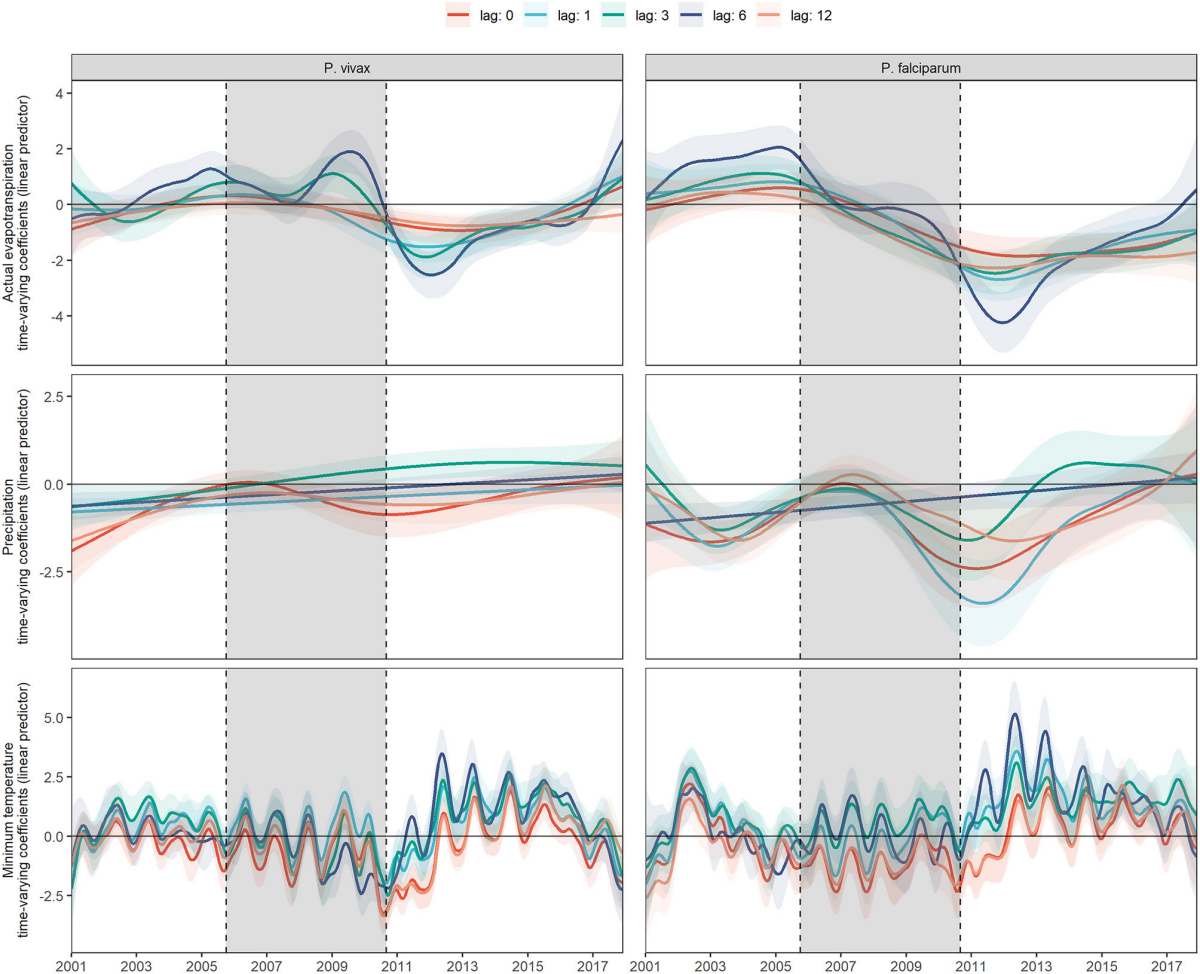
Fitted time-varying meteorological effects on MIR by parasite species at different time lags. Fitted time-varying meteorological effects at different time lags and their 95% CI (shading of each colored curve). The period shaded in gray represents the PAMAFRO intervention.

Figure 3 shows the fitted coefficients and their 95% confidence interval for the meteorological variables lagged by different numbers of months. Different models were fitted for each lag value. The magnitude and direction of the lagged effects of the meteorological variables on each parasite species changed differently throughout the study, although most of the lags for each variable follow a similar trend. In the case of actual evapotranspiration, its 6-month lagged effects on *P. vivax* and *P. falciparum* intensified more over time compared to the other lags. For *P. vivax*, it was exacerbated toward a more positive effect between 2008 and 2010, following an exacerbation to a more negative effect from 2010 to 2013. For *P. falciparum*, it transitioned from null effects most of the time during the intervention to a more negative effect after the PAMAFRO intervention between 2010 and 2013.

For precipitation, the exacerbation to a more negative effect at the end of PAMAFRO was observed for the no lagged and 12-month lagged effects on *P. vivax*, and all the lagged effects, except the 6-month lagged effect on *P. falciparum*. Finally, for the minimum temperature, all the lagged effects suggest evidence of a seasonal pattern. The greatest intensification was observed on the 6-month lagged effect after the PAMAFRO intervention, from 2012 to 2014, where the effects on each parasite species increased in magnitude toward a more positive effect.

Model performance metrics were presented in Supplementary Table 2. For *P. vivax*, the 1-, 3-, and 6-month lagged covariates models performed better for the AIC. For instance, the 6-month lag had the best AIC value, and the 12-month lagged covariates model had the worst. The more complex model was the 3-month lagged covariates model, as it had higher degrees of freedom, and, conversely, the simpler one was the 1-month lagged covariates model. For *P. falciparum*, results were similar, but the base model, in this case, was slightly better than the 3-month lagged covariates model. The more complex model for *P. falciparum* was the model with 6-month lags, and the simpler one was the model with a 12-month lag.

## Discussion

By analyzing panel data of the Peruvian Amazon from 2001 to 2017, this study found a critical disruption in the baseline relationship between malaria and climate after the interruption of the malaria control period implemented by PAMAFRO. At the end of the PAMAFRO intervention, the estimated effects (dose-response) of actual evapotranspiration, precipitation, and minimum temperature on the incidence rates decreased for both malaria parasite species. Although this decrease diminished after ~2 years, the dose-response effects did not return to baseline levels (before the PAMAFRO period). Notably, the interruption of the PAMAFRO project impacted the time-varying effects of meteorological variables such as precipitation and actual evapotranspiration more markedly for *P. falciparum* compared with *P. vivax*. These findings highlight the importance of sustaining malaria control efforts since interruption may destabilize the baseline (pre-PAMAFRO) associations between meteorological factors and malaria incidence rates. Also, the time-varying effect of meteorological factors on malaria incidence should be used to inform and update EWS in the context of climate change projections.

The effect of meteorological variables on malaria incidence, when the PAMAFRO intervention was discontinued, demonstrated that the relationship between malaria and climate intensified in a region of low malaria incidence. This emphasizes the importance of monitoring meteorological variation during the elimination process to detect early disruptions of this relationship. EWS play a pivotal role in this context when considering these time-varying effects. Previous studies have revealed the importance of implementing integrated surveillance and control systems for monitoring meteorological indicators in predicting abrupt changes in the risk of malaria in hotspots of the Amazon region (46).

The effect of meteorological variables on *P. falciparum* was longer and temporally significant compared with *P. vivax* where the decreases are potentially related to activation of the silent latent parasite stage (hypnozoite), that results in relapses (47). In addition, *P. falciparum* has been associated with minimum temperatures over time (14, 48), due to its shorter optimal developmental temperature range (15°-30°C) compared to *P. vivax* (18°-30°C) (49). High temperature shortens the parasite development inside the mosquito; thus the EIP is ~9–15 days for *P. falciparum* and 8–24 days for *P. vivax* (50).

Our results were consistent with previous studies in terms of heterogeneity among meteorological variables. A predictive study reported that moderate temperature (23–24°C) was related to the highest risk of malaria at a lag of 2 weeks, with a significantly increased risk up to 4 weeks of lag (51). Intriguingly, this is consistent with the incubation period of the parasite inside a human host; the time elapsed between the mosquito bite and the manifestation of symptoms is ~9–14 days for *P. falciparum* and 12–17 days for *P. vivax* infection (52). The parasite EIP inside the mosquito ranges between 1 and 3 weeks (50), reinforcing the seasonal relationship between temperature and malaria incidence. In addition, our study described a significantly longer effect of precipitation and evapotranspiration on malaria incidence; this could be explained by the formation of optimal mosquito breeding sites as a consequence of rainfall over the previous year (51). The development of natural breeding sources is a long-term process that depends not only on precipitation but also on vegetation and soil moisture (which control evapotranspiration) (53). In other vector-borne diseases such as dengue, there was a similar pattern of meteorological variables: the minimum temperature (26°C) with 0–5 weeks lag and precipitation (60 mm) with 8–15 weeks lag was related with increased dengue incidence (54).

Our findings are relevant for current and future climate change. Climate change causes unexpected circumstances such as land degradation and disruption, floods, and droughts that destabilize the economic growth, population health and often increase migration (23, 55), which alter the operation of control activities, as well as interactions among the environment, mosquito, and host. Mosquitoes circulate in impacted environments near human settlements, resulting in increased malaria risk (56). Also, rising global temperature is a major contributor to the increasing spread of malaria even in non-endemic areas (57, 58). In addition, governments need to monitor and enforce the implementation of malaria interventions (59). In Zambia, for example, many of the positive effects of control programs are being reduced by the negative impact of climatic conditions that favor the spread of malaria (60). These scenarios may lead to a more substantial resurgence, changes in seasonal trends, and complications for malaria control and elimination.

After a period of low malaria rates due to control interventions, malaria re-emerged due to weakening control activities, technical problems, and increasing malaria potential (61). Funding constraints are the most common cause of control program interruptions. After only 4 months of discontinuing malaria prevention activities in Uganda, malaria cases increased (62). In Venezuela, the high incidence rates of malaria continue to increase due to the catastrophic socio-economic and political crisis that decimated vector-borne disease control programs. Prolonged poverty, lack of hygiene, and malnutrition has increased population-level susceptibility to infectious diseases (63). A major contributing factor has been the massive migration of Venezuelans to neighboring countries resulting in the importation of malaria cases to border regions (64). Similar situations have been reported around the world (11, 12, 61). Despite decreases in funding from international entities and global funds, we strongly recommend that intervention be considered an ongoing and necessary expense (65). Effective control of malaria hotspots could reduce malaria mortality and morbidity (66). Changes in land cover due to human settlement may also alter the temperature and increase mosquito migration to non-endemic high altitudes, for example in Kenya (67). One study showed that mosquitoes carried by wind continued surviving, breeding, and feeding on human blood, potentially expanding malaria transmission to new altitudes (68).

We acknowledge the following limitations to this study. First, passive case detection data are recorded only for individuals overwhelmed by symptoms who seek care at health services; health personnel do not conduct active case detection in the community. Hence the results of these findings are relevant only for clinical cases, and further studies are required to understand whether the same patterns are observed for asymptomatic cases, which contribute to the maintenance of parasite transmission (69). Second, limited access to remote areas in the Amazon region prevents continuous health care access to indigenous communities, thus the malaria caseload is an underestimate. Low geographic accessibility and travel time have been found to be barriers to reaching remote health facilities (35). Third, some reports have indicated that mosquito activities such as host-seeking behavior were influenced by wind speed affecting human CO_2_ dispersion, and other studies reported that the mosquito movement depends on wind direction (70). However, we did not include other meteorological variables such as wind speed in this analysis. Instead, we selected meteorological variables commonly used in epidemiological malaria studies. Finally, other factors such as natural disasters or political crises were not considered for this analysis; to the best of our knowledge, no major events were reported in this area during the study period.

## Conclusions

The sustainability of malaria elimination efforts deserves particular attention due to current climate change challenges. This study provided evidence of important deviations of the baseline climate-malaria trends concurrent with the interruption of malaria control interventions in the study area. These disruptions significantly affect the transmission of *P. falciparum* in comparison to *P. vivax* and provide information for tailoring future malaria control activities.

## Data Availability Statement

The data analyzed in this study is subject to the following licenses/restrictions: Dataset will be available upon request to the Peruvian Ministry of Health. Requests to access these datasets should be directed to transparencia@minsa.gob.pe.

## Ethics Statement

Ethical review and approval was not required for the study on human participants in accordance with the local legislation and institutional requirements. Written informed consent from the participants' legal guardian/next of kin was not required to participate in this study in accordance with the national legislation and the institutional requirements.

## Author Contributions

GC-E conceived the idea of the study. GC-E, JQ, DV, and RC designed the study. DV and RC analyzed the data. GC-E wrote the article with contributions from JQ, DV, RC, AL-C, and TB. All authors contributed to the article and approved the submitted version.

## Conflict of Interest

The authors declare that the research was conducted in the absence of any commercial or financial relationships that could be construed as a potential conflict of interest.

## Publisher's Note

All claims expressed in this article are solely those of the authors and do not necessarily represent those of their affiliated organizations, or those of the publisher, the editors and the reviewers. Any product that may be evaluated in this article, or claim that may be made by its manufacturer, is not guaranteed or endorsed by the publisher.

## References

[1] World Health Organization. World Malaria Report 2020. (2020). Available online at: https://www.who.int/teams/global-malaria-programme/reports/world-malaria-report-2020 (accessed: February 7, 2021).

[2] Centro Nacional de Epidemiología Prevención y Control de Enfermedades. Boletín Epidemiológico del Perú - SE 42. (2019). Available online at: https://www.dge.gob.pe/portal/docs/vigilancia/boletines/2019/42.pdf (accessed on: December 1, 2020).

[3] Soto-Calle V Rosas-Aguirre A Llanos-Cuentas A Abatih E Dedeken R Rodriguez H. Spatio-temporal analysis of malaria incidence in the Peruvian Amazon Region between 2002 and 2013. Sci Rep. (2017) 7:40350. 10.1038/srep40350 28091560 PMC5238441

[4] Rosas-Aguirre A Gamboa D Manrique P Conn JE Moreno M Lescano AG. Epidemiology of *Plasmodium vivax* malaria in Peru. Am J Trop Med Hyg. (2016) 95:133–44. 10.4269/ajtmh.16-0268 27799639 PMC5201219

[5] Chowell G Munayco CV Escalante AA McKenzie FE. The spatial and temporal patterns of falciparum and vivax malaria in Peru: 1994–2006. Malar J. (2009) 8:142. 10.1186/1475-2875-8-142 19558695 PMC2714521

[6] Rosas-Aguirre A Guzmán-Guzmán M Moreno-Gutierrez D Rodriguez-Ferrucci H Vargas-Pacherrez D González A. Long-lasting insecticide - treated bednet ownership, retention and usage one year after their distribution in Loreto, Peru. Rev Peru Med Exp Salud Publica. (2011) 28:228–64. 10.1590/S1726-46342011000200009 21845302

[7] Rosas-Aguirre A Gamboa D Rodriguez H Llanos-Zavalaga F Aguirre K Llanos-Cuentas A. Use of standardized blood smear slide sets for competency assessment in the malaria microscopic diagnosis in the Peruvian Amazon. Rev Peru Med Exp Salud Publica. (2010) 27:540–7. 10.1590/S1726-46342010000400008 21308193

[8] Rosas-Aguirre A. Understanding transmission dynamics for malaria control and elimination in peru [Doctoral Dissertation]. Université catholique de Louvain, Brussels, Belgium (2015). Available online at: https://dial.uclouvain.be/pr/boreal/object/boreal%3A165403/datastream/PDF_01/view (accessed March 10, 2021).

[9] Rosas-Aguirre A Speybroeck N Llanos-Cuentas A Rosanas-Urgell A Carrasco-Escobar G Rodriguez H. Hotspots of malaria transmission in the Peruvian amazon: rapid assessment through a parasitological and serological survey. PLoS ONE. (2015) 10:e0137458. 10.1371/journal.pone.0137458 26356311 PMC4565712

[10] Bennett A Yukich J Miller JM Keating J Moonga H Hamainza B. The relative contribution of climate variability and vector control coverage to changes in malaria parasite prevalence in Zambia 2006–2012. Parasit Vectors. (2016) 9:431. 10.1186/s13071-016-1693-0 27496161 PMC4974721

[11] Aregawi MW Ali AS Al-Mafazy AW Molteni F Katikiti S Warsame M. Reductions in malaria and anaemia case and death burden at hospitals following scale-up of malaria control in Zanzibar, 1999–2008. Malar J. (2011) 10:46. 10.1186/1475-2875-10-46 21332989 PMC3050777

[12] Mharakurwa S Thuma PE Norris DE Mulenga M Chalwe V Chipeta J. Malaria epidemiology and control in Southern Africa. Acta Trop. (2012) 121:202–6. 10.1016/j.actatropica.2011.06.012 21756864 PMC3214248

[13] Mosnier E Dusfour I Lacour G Saldanha R Guidez A Gomes MS. Resurgence risk for malaria, and the characterization of a recent outbreak in an Amazonian border area between French Guiana and Brazil. BMC Infect Dis. (2020) 20:373. 10.1186/s12879-020-05086-4 32456698 PMC7249302

[14] Fletcher IK Stewart-Ibarra AM Sippy R Carrasco-Escobar G Silva M Beltran-Ayala E. The relative role of climate variation and control interventions on malaria elimination efforts in El Oro, Ecuador: a modeling study. Front Environ Sci. (2020) 8:135. 10.3389/fenvs.2020.00135

[15] Thomson MC Muñoz ÁG Cousin R Shumake-Guillemot J. Climate drivers of vector-borne diseases in Africa and their relevance to control programmes. Infect Dis Poverty. (2018) 7:81. 10.1186/s40249-018-0460-1 30092816 PMC6085673

[16] Castro MC. Malaria transmission and prospects for malaria eradication: the role of the environment. Cold Spring Harb Perspect Med. (2017) 7:a025601. 10.1101/cshperspect.a025601 28490534 PMC5629986

[17] Brugueras S Fernández-Martínez B Martínez-de la Puente J Figuerola J Porro TM Rius C. Environmental drivers, climate change and emergent diseases transmitted by mosquitoes and their vectors in southern Europe: a systematic review. Environ Res. (2020) 191:110038. 10.1016/j.envres.2020.110038 32810503

[18] Christiansen-Jucht CD Parham PE Saddler A Koella JC Basáñez MG. Larval and adult environmental temperatures influence the adult reproductive traits of *Anopheles gambiae s.s*. Parasit Vect. (2015) 8:456. 10.1186/s13071-015-1053-5 26382035 PMC4573685

[19] Parham PE Pople D Christiansen-Jucht C Lindsay S Hinsley W Michael E. Modeling the role of environmental variables on the population dynamics of the malaria vector *Anopheles gambiae* sensu stricto. Malar J. (2012) 11:271. 10.1186/1475-2875-11-271 22877154 PMC3496602

[20] Paaijmans KP Wandago MO Githeko AK Takken W. Unexpected high losses of *Anopheles gambiae* larvae due to rainfall. PLoS ONE. (2007) 2:1146. 10.1371/journal.pone.0001146 17987125 PMC2063461

[21] Zeilhofer P dos Santos ES Ribeiro ALM Miyazaki RD dos Santos MA. Habitat suitability mapping of *Anopheles darlingi* in the surroundings of the Manso hydropower plant reservoir, Mato Grosso, Central Brazil. Int J Health Geogr. (2007) 6:7. 10.1186/1476-072X-6-7 17343728 PMC1851006

[22] Le PVV Kumar P Ruiz MO Mbogo C Muturi EJ. Predicting the direct and indirect impacts of climate change on malaria in coastal Kenya. PLoS ONE. (2019) 14:e0211258. 10.1371/journal.pone.0211258 30726279 PMC6364917

[23] Rossati A Bargiacchi O Kroumova V Zaramella M Caputo A Garavelli Pietro L. Climate, environment and transmission of malaria. Infez Med. (2016) 24:93–104. 27367318

[24] Patz JA Olson SH. Malaria risk and temperature: influences from global climate change and local land use practices. Proc Natl Acad Sci USA. (2006) 103:5635–6. 10.1073/pnas.0601493103 16595623 PMC1458623

[25] Guo C Yang L Ou CQ Li L Zhuang Y Yang J. Malaria incidence from 2005–2013 and its associations with meteorological factors in Guangdong, China. Malar J. (2015) 14:116. 10.1186/s12936-015-0630-6 25881185 PMC4389306

[26] White MT Shirreff G Karl S Ghani AC Mueller I. Variation in relapse frequency and the transmission potential of *Plasmodium vivax* malaria. Proc R Soc B Biol Sci. (2016) 283:20160048. 10.1098/rspb.2016.0048 27030414 PMC4822465

[27] Semenza JC Suk JE. Vector-borne diseases and climate change: a European perspective. FEMS Microbiol Lett. (2018) 365:244. 10.1093/femsle/fnx244 29149298 PMC5812531

[28] Maharaj R. Early warning systems for the detection of malaria outbreaks. Indian J Med Res. (2017) 146:560–2. 10.4103/ijmr.IJMR_933_17 29512597 PMC5861466

[29] Teklehaimanot HD Schwartz J Teklehaimanot A Lipsitch M. Weather-based prediction of *Plasmodium falciparum* malaria in epidemic-prone regions of Ethiopia II. Weather-based prediction systems perform comparably to early detection systems in identifying times for interventions. Malar J. (2004) 3:44. 10.1186/1475-2875-3-44 15555061 PMC535541

[30] Dureau J Kalogeropoulos K Baguelin M. Capturing the time-varying drivers of an epidemic using stochastic dynamical systems. Biostatistics. (2013) 14:541–55. 10.1093/biostatistics/kxs052 23292757

[31] Ministerio de Desarrollo e Inclusión Social. Reporte regional de indicadores sociales del departamento de Loreto. (2020). Available online at: http://sdv.midis.gob.pe/RedInforma/Reporte/ReportePDF?vCodTema=1 (accessed on: December 18, 2020).

[32] Instituto Nacional de Estadística e Informática. Compendio Estadístico: Loreto 2017. (2017). Available online at: https://www.inei.gob.pe/media/MenuRecursivo/publicaciones_digitales/Est/Lib1501/libro.pdf (accessed on: December 18, 2020).

[33] Instituto Nacional de Estadística e Informática. Población afiliada a algún seguro de salud. (2018). Available online at: http://censo2017.inei.gob.pe/publicaciones_especiales/ (accessed: December 10, 2020).

[34] Brierley CK Suarez N Arora G Graham D. Healthcare access and health beliefs of the indigenous peoples in remote amazonian Peru. Am J Trop Med Hyg. (2014) 90:180–3. 10.4269/ajtmh.13-0547 24277789 PMC3886418

[35] Carrasco-Escobar G Manrique E Tello-Lizarraga K Miranda JJ. Travel time to health facilities as a marker of geographical accessibility across heterogeneous land coverage in Peru. Front Public Health. (2020) 8:498. 10.3389/fpubh.2020.00498 33042942 PMC7524891

[36] Ministerio del ambiente. SENAMHI - Loreto (2020). Available online at: https://www.senamhi.gob.pe/main.php?dp=loreto&p=pronostico-detalle (accessed on: January 31, 2021)

[37] Ministerio de Salud. Herramientas Para la Vigilancia Epidemiológica. (2005). Available online at: https://www.dge.gob.pe/portalnuevo/publicaciones/materiales/herramientas-para-la-vigilancia-epidemiologica/ (accessed on: January 27, 2021).

[38] Ministerio de Salud. Directiva sanitaria N° 046 - *MINSA/DGE-V.01 de notificación de enfermedades y eventos sujetos a vigilancia epidemiológica en salud pública*. (2013). Available online at: https://www.gob.pe/institucion/minsa/informes-publicaciones/280839-directiva-sanitaria-n-046-minsa-dge-v-01-de-notificacion-de-enfermedades-y-eventos-sujetos-a-vigilancia-epidemiologica-en-salud-publica (accessed: January 27, 2021).

[39] Instituto Nacional de Salud. Manual de procedimientos de laboratorio para el diagnóstico de malaria. (2003). Available online at: https://www.gob.pe/institucion/minsa/informes-publicaciones/353447-manual-de-procedimientos-de-laboratorio-para-el-diagnostico-de-malaria (accessed: January 27, 2021).

[40] Ministerio de Salud. Norma técnica de salud para la atención de la malaria y malaria grave en el Perú. (2015). Available online at: https://www.gob.pe/institucion/minsa/informes-publicaciones/280813-norma-tecnica-de-salud-para-la-atencion-de-la-malaria-y-malaria-grave-en-el-peru (accessed: January 27, 2021).

[41] Pan American Health Organization. Compartiendo Lecciones Aprendidas. Proyecto control de la Malaria en las zonas fronterizas de la Región Andina: Un enfoque comunitario - PAMAFRO (2011). Available online at: https://www.paho.org/es/documentos/compartiendo-lecciones-aprendidas-proyecto-control-malaria-zonas-fronterizas-region (accessed on: January 25, 2021).

[42] Abatzoglou JT Dobrowski SZ Parks SA Hegewisch KC. TerraClimate, a high-resolution global dataset of monthly climate and climatic water balance from 1958-2015. Sci Data. (2018) 5:170191. 10.1038/sdata.2017.191 29313841 PMC5759372

[43] Bakdash JZ Marusich LR. Repeated measures correlation. Front Psychol. (2017) 8:456. 10.3389/fpsyg.2017.00456 28439244 PMC5383908

[44] Wood SN. Fast stable restricted maximum likelihood and marginal likelihood estimation of semiparametric generalized linear models. J R Stat Soc Ser B (Statistical Methodol. (2011) 73:3–36. 10.1111/j.1467-9868.2010.00749.x

[45] Wood SN. Generalized Additive Models: An Introduction With R. 2nd ed. Chapman and Hall/CRC (2017). 10.1201/9781315370279

[46] Ruiz D Ceroń V Molina AM Quiñones ML Ji?enez MM Ahumada M. Implementation of malaria dynamic models in municipality level early warning systems in Colombia. Part I: description of study sites. Am J Trop Med Hyg. (2014) 91:27–38. 10.4269/ajtmh.13-0363 24891460 PMC4080564

[47] White NJ. Determinants of relapse periodicity in *Plasmodium vivax* malaria. Malar J. (2011) 10:297. 10.1186/1475-2875-10-297 21989376 PMC3228849

[48] Bi Y Yu W Hu W Lin H Guo Y Zhou XN. Impact of climate variability on *Plasmodium vivax* and *Plasmodium falciparum* malaria in Yunnan Province, China. Parasit Vectors. (2013) 6:357. 10.1186/1756-3305-6-357 24341555 PMC3898806

[49] Olliaro PL Barnwell JW Barry A Mendis K Mueller I Reeder JC. Implications of *Plasmodium vivax* biology for control, elimination, and research. Am J Trop Med Hyg. (2016) 95:4–14. 10.4269/ajtmh.16-0160 27799636 PMC5201222

[50] Thomas S Ravishankaran S Justin NAJA Asokan A Kalsingh TMJ Mathai MT. Microclimate variables of the ambient environment deliver the actual estimates of the extrinsic incubation period of *Plasmodium vivax* and *Plasmodium falciparum*: a study from a malaria-endemic urban setting, Chennai in India. Malar J. (2018) 17:201. 10.1186/s12936-018-2342-1 29769075 PMC5956829

[51] Kim Y Ratnam JV Doi T Morioka Y Behera S Tsuzuki A. Malaria predictions based on seasonal climate forecasts in South Africa: a time series distributed lag nonlinear model. Sci Rep. (2019) 9:17882. 10.1038/s41598-019-53838-3 31784563 PMC6884483

[52] Warrell DA Gilles HM. Essential Malariology. 4th ed. New York, NY: Oxford University Press (2002).

[53] Malahlela OE Olwoch JM Adjorlolo C. Evaluating efficacy of landsat-derived environmental covariates for predicting malaria distribution in rural villages of Vhembe District, South Africa. Ecohealth. (2018) 15:23–40. 10.1007/s10393-017-1307-0 29330677

[54] Kakarla SG Caminade C Mutheneni SR Morse AP Upadhyayula SM Kadiri MR. Lag effect of climatic variables on dengue burden in India. Epidemiol Infect. (2019) 147:e170. 10.1017/S0950268819000608 31063099 PMC6518529

[55] Arora NK. Impact of climate change on agriculture production and its sustainable solutions. Environ Sustain. (2019) 2:95–6. 10.1007/s42398-019-00078-w

[56] Alimi TO Fuller DO Qualls WA Herrera SV Arevalo-Herrera M Quinones ML. Predicting potential ranges of primary malaria vectors and malaria in northern South America based on projected changes in climate, land cover and human population. Parasit Vectors. (2015) 8:431. 10.1186/s13071-015-1033-9 26289677 PMC4546039

[57] Moukam Kakmeni FM Guimapi RYA Ndjomatchoua FT Pedro SA Mutunga J Tonnang HEZ. Spatial panorama of malaria prevalence in Africa under climate change and interventions scenarios. Int J Health Geogr. (2018) 17:2. 10.1186/s12942-018-0122-3 29338736 PMC5771136

[58] Dhimal M Ahrens B Kuch U. Climate change and spatiotemporal distributions of vector-borne diseases in Nepal - A systematic synthesis of literature. PLoS ONE. (2015) 10:e0129869. 10.1371/journal.pone.0129869 26086887 PMC4472520

[59] Gething PW Smith DL Patil AP Tatem AJ Snow RW Hay SI. Climate change and the global malaria recession. Nature. (2010) 465:342–5. 10.1038/nature09098 20485434 PMC2885436

[60] Nawa M Halwindi H Hangoma P. Modelling malaria reduction in a highly endemic country: Evidence from household survey, climate, and programme data in Zambia. J Public Health Afr. (2020) 11:1096. 10.4081/jphia.2020.1096 33209231 PMC7649733

[61] Cohen JM Smith DL Cotter C Ward A Yamey G Sabot OJ. Malaria resurgence: a systematic review and assessment of its causes. Malar J. (2012) 11:122. 10.1186/1475-2875-11-122 22531245 PMC3458906

[62] Raouf S Mpimbaza A Kigozi R Sserwanga A Rubahika D Katamba H. Resurgence of malaria following discontinuation of indoor residual spraying of insecticide in an area of Uganda with previously high-transmission intensity. Clin Infect Dis. (2017) 65:453–60. 10.1093/cid/cix251 28369387 PMC5850037

[63] Grillet ME Hernández-Villena JV Llewellyn MS Paniz-Mondolfi AE Tami A Vincenti-Gonzalez MF. Venezuela's humanitarian crisis, resurgence of vector-borne diseases, and implications for spillover in the region. Lancet Infect Dis. (2019) 19:e149–61. 10.1016/S1473-3099(18)30757-6 30799251

[64] Arisco NJ Peterka C Castro MC. Cross-border malaria in Northern Brazil. Malar J. (2021) 20:135. 10.1186/s12936-021-03668-4 33676522 PMC7937307

[65] Shretta R Avanceña ALV Hatefi A. The economics of malaria control and elimination: a systematic review. Malar J. (2016) 15:593. 10.1186/s12936-016-1635-5 27955665 PMC5154116

[66] Scott N Hussain SA Martin-Hughes R Fowkes FJI Kerr CC Pearson R. Maximizing the impact of malaria funding through allocative efficiency: using the right interventions in the right locations. Malar J. (2017) 16:368. 10.1186/s12936-017-2019-1 28899373 PMC5596957

[67] Kweka EJ Kimaro EE Munga S. Effect of deforestation and land use changes on mosquito productivity and development in western Kenya highlands: Implication for malaria risk. Front Public Health. (2016) 4:238. 10.3389/fpubh.2016.00238 27833907 PMC5080343

[68] Sanogo ZL Yaro AS Dao A Diallo M Yossi O Samaké D. The effects of high-altitude windborne migration on survival, oviposition, and blood-feeding of the African malaria mosquito, *Anopheles gambiae* s.l. (Diptera: Culicidae). J Med Entomol. (2020) 58:343–9. 10.1093/jme/tjaa137 32667040 PMC7801746

[69] Cheaveau J Mogollon DC Mohon MAN Golassa L Yewhalaw D Pillai DR. Asymptomatic malaria in the clinical and public health context. Expert Rev Anti Infect Ther. (2019) 17:997–1010. 10.1080/14787210.2019.1693259 31718324

[70] Endo N Eltahir EAB. Modelling and observing the role of wind in *Anopheles* population dynamics around a reservoir. Malar J. (2018) 17:48. 10.1186/s12936-018-2197-5 29370803 PMC5784732

