# Supplementary material for: Time-Varying Effects of Meteorological Variables on Malaria Epidemiology in the Context of Interrupted Control Efforts in the Amazon Rainforest, 2000–2017
Source: Front Med (Lausanne). 2021 Sep 29;8:721515. doi: 10.3389/fmed.2021.721515 (PMC8511324; doi:10.3389/fmed.2021.721515)
Supplement: Supplementary file 1 [file Data_Sheet_1.PDF]

Supplementary Material

1 Supplementary Methods

1.1 Repeated measures correlation

Repeated measures correlation (1) is a technique based on the Analysis of covariance (ANCOVA) to calculate a sample (Pearson) correlation coefficient that captures the common intra-individual linear association of two observed variables. When having repeated measurements over individuals for some variables, the common approach to calculate a correlation coefficient between a pair of them is first aggregate the observations using the average over the individuals, or just calculating the correlation coefficient over all the data. These approaches can lead to spurious correlation coefficients that can lead to wrong conclusions about the association between the variables if a graphical checking is not done priorly. Having a technique that accounts for the intra-individual association, isolating the effect of the repeated measurements over the individuals, is recommended to correctly assess the association. The approach for constructing this sample statistic can be thought as constructing a null multilevel model with different intercepts for each individual and the same slope to represent the linear association between the observed variables. However, the repeated measures correlation method only accounts for the intra-individual variability in the observations, while multilevel modelling accounts for different sources of variation. Assuming there is a linear association between the observed variables, ANCOVA is used to isolate the variability explained by the individuals and the variability explained by the covariate over all the individuals. The latter is then used to calculate the repeated measures correlation coefficient as the squared of the proportion of the total intra-variability of the response (having removed the inter-individual variability) that is explained by the covariate. The sign of the repeated measures correlation coefficient corresponds to the sign of the common slope of the linear regression line that describes the association between the observed variables. The main limitation of this technique is that it only assesses linear associations, as the Pearson correlation coefficient does. It is recommended to aid the analysis plotting the data. Package ***rmcorr*** in R (*https://cran.r-project.org/web/packages/rmcorr/rmcorr.pdf)* implements the procedure to estimate the repeated measures correlation coefficient between two variables indicating the variable in the data that represent the individuals/groups. In addition, it includes a scatterplot function to visually inspect the adjusted linear regression lines at each individual/group level with different colors. In our study, the districts were used as individuals/groups to analyze the correlation between the meteorological variables.

1.2 Distribution assumptions

The election of the Negative Binomial (NB) distribution for the monthly counts of new malaria cases by each of the parasite species in the districts was made to account for the over-dispersion in the data. Another concern was the percentage of zero counts in the data. There are districts that have a serious number of months with zero count of malaria cases, as is the case of most of the districts in the province of Ucayali. Zero counts represent almost 30% of the whole data, which is not expected for the theoretical distribution assumed. Currently, the mgcv package allows to fit zero-inflated or hurdle Poisson regression models but choosing these distributions will not address the over-dispersion problem, which is more likely to further affect the quality of fit of the models. Hence, opted for the negative binomial distribution.

Supplementary Material should be uploaded separately on submission. Please include any supplementary data, figures and/or tables. All supplementary files are deposited to FigShare for permanent storage and receive a DOI.

1.3 Generalized Additive Models

Generalized additive models (GAMs) (2) represent a more flexible option than the widely used *generalized linear models* (3), as they allow including in the linear predictor both non-parametric terms (smooth functions) and the usual parametric and polynomial terms to express the form of the effect of each covariate in the model. In general, regression models where the coefficient of each of the linear effect term of a set of covariates is expressed as a function over some other variables are called *varying-coefficients models* (4). Here, the coefficients vary over time, hence the name of *time-varying coefficients models*. GAMs allow us to further specify the coefficients as smooth functions over time, meaning that the coefficients are mapped from the range of time points in a continuous way.

In a classical regression setting, the linear effect of the j-th covariate measured at time t and district i would be represented by $\beta_j x_{ijt}$, where β_j is the regression coefficient to be estimated. Notice that a single value of β_j is estimated for each covariate without regard to the time point, as it is indexed only over the number of covariates. To analyze how the magnitude and direction of the coefficients vary over the set of time points, these coefficients are expressed as functions of time, that is $f_j(t)x_{ijt}$, where f_j now denotes the coefficient. Furthermore, these functions are smooth over time –a continuous shape over the range of time points. The **mgcv** package allows to estimate these smooth functions using *penalized regression splines*. The splines consist in a linear combination of a set of *basis functions*. Hence, the smooth functions over time are written as

$$f_j(t) = \sum_{k=1}^{K} \varphi_{jk} b_{jk}(t),$$

where b_{jk} denotes the k-th basis function, φ_{jk} denotes a coefficient for the k-th basis function in the linear combination, and K is the settled number of basis functions, called the *basis dimension*. The basis dimension controls the complexity of the smoother. The bigger the number of basis functions, the more flexible and complex the form of the smooth function. A penalty term is imposed in the fitting procedure to account for the degree of complexity of each smooth function. The form of penalty term depends on the election of the splines (basis functions), and the amount of penalty is controlled by a *smoothing parameter*. Given the smoothing parameter, fitting is made using *penalized iteratively reweighted least squats* (PIRLS). To estimate the smoothing parameter so it optimizes the likelihood of the model, package **mgcv** offers an implementation of the restricted maximum likelihood (REML) estimation algorithm done by Wood (2011) (5), as well as other estimation algorithms. This implementation of REML provides some improvements in the accuracy and stability of the smoothing parameter estimates at a computation cost comparable to other algorithms in the package. Given the estimated smoothing parameters, the *effective degrees of freedom* (EDF) were calculated for each smooth function, which represents the complexity of the smoother after penalization. The estimation of the scale parameter of the NB distribution is also done using REML alongside the estimation of the smoothing parameters. Finally, as data is smoothed only over time, there is no need to specify more than one type of spline. The default and more recommended option in package **mgcv** are the *thin plate regression splines* (TPRS) (6). The main advantages of TPRS are that it avoids knot placement for constructing the basis and works efficiently with large data sets, as it is our case. They also have others

desirable features, like rotational invariance and low rank approximation, which can be further revisited in Wood (2003) (6) and Wood (2017) (7).

As it is asserted by Wood (2017) (7), there is an equivalence between Gaussian random effects and smooth terms when using REML, so the formers can be estimated using the methods described above for smooth terms. In package **mgcv** this can be done by specifying a smooth term for the districts with a "random effects" smoothing basis.

1.4 Model fitting

Model diagnosis was done for each fitted model, which consisted of revisiting classical diagnostic plots and running a simulation-based test to check for the adequacy of the basis dimensions. Both of these diagnoses were produced by the **gam.check** function on package **mgcv**. To check the adequacy of the basis dimensions, the base model was fitted with the default basis dimension, which is 10. According to the results of the test, this dimension was too low for all the smooth terms. An iterative process resulted in a basis dimension 50 for the smooth term corresponding to actual evapotranspiration, 30 for precipitation, and 100 for minimum temperature. For the smooth "random effect" term of the districts, we set the basis dimension to 49, as there were 49 districts.
It is worth mentioning that, in the GAMs literature, when a smooth term can be approximated by other smooth terms in a model cause a problem of concurvity. Extremely high levels of concurvity can cause problems of identifiability of smooth terms, as well as instability in the estimations. The **concurvity** function in the **mgcv** package can be taught as a generalized form of multicollinearity. This function produces a set of indices that range from 0 to 1 to check the degree of concurvity on the model. A value of 0 means no problem, and a value of 1 means a total lack identifiability between the smooth terms. Now, as all the smooth terms in our models are specified over time, our models were conditioned to have high levels of concurvity. Furthermore, concurvity can also arise if two or more covariates included in the model are high-correlated, as a multicollinearity problem. Therefore, all the fitted models were carefully inspected for concurvity, and their estimated coefficients and confidence intervals visually analyzed to inspect for serious distortions.

1.5 Construction of the time measurements for GAM

For constructing the time variable, the smoothers were used as input and a date-time variable was created using the year of the month of the observations and setting the day to the first day of the month and the time at 00:00:00 UTC. We then converted this date-time values into integers. These integers correspond to the Unix timestamp (number of seconds from January 1st, 1970). An important detail is that, to make the models comparable, we filter out the observations of the first year on the period of study, that is, 2000. This was done because for the 12 month lagged variables on this year, we do not have observations for the previous year to lag on, so we have to start at year 2001 to be able to use the observations in 2000 as lagged values. Consequently, the data for model building starts from January 2001.

2 Supplementary Tables

2.1 Supplementary Table 1.

Supplementary Table 1. Repeated measures correlation values (r) with their 95% confidence interval (CI) for the meteorological variables

Climate variable	1		2		3		4		5	
	r	**95% CI**	***r***	**95% CI**	***r***	**95% CI**	***r***	**95% CI**	***r***	**95% CI**
1 Actual evapotranspiration	–									
2 Precipitation	-0.19	[-0.21, -0.17]	–							
3 Runoff	-0.31	[-0.33, -0.30]	0.99	[0.99, 0.99]	–					
4 Maximum temperature	0.56	[0.55, 0.57]	-0.19	[-0.21, -0.17]	-0.25	[-0.23, -0.27]	–			
5 Minimum temperature	0.06	[0.04, 0.08]	0.17	[0.15, 0.19]	0.15	[0.13, 0.17]	0.44	[0.43, 0.46]	–	

Supplementary Table 2. Degrees of freedom (DF) and Akaike Information Criterion (AIC) values for the models with different time lags by parasite species

Model	*P. vivax*		*P. falciparum*	
	**DF**	**AIC**	**DF**	**AIC**
No lag	130.38	70783.24	122.05	46213.92
1 month lag	128.30	70755.05	124.56	46174.96
3 month lag	137.48	70763.09	123.03	46216.87
6 month lag	133.49	70689.94	127.79	46137.46
12 month lag	128.86	70807.74	118.56	46221.94

3 Supplementary Figures

Supplementary Figure 1. 6-month moving averages of the meteorological variables from 2000 to 2017 in the province of Alto Amazonas. Grey lines: Observed measurements at a district level. Colored lines: 6-month moving averages over the observed measurements at a district level.

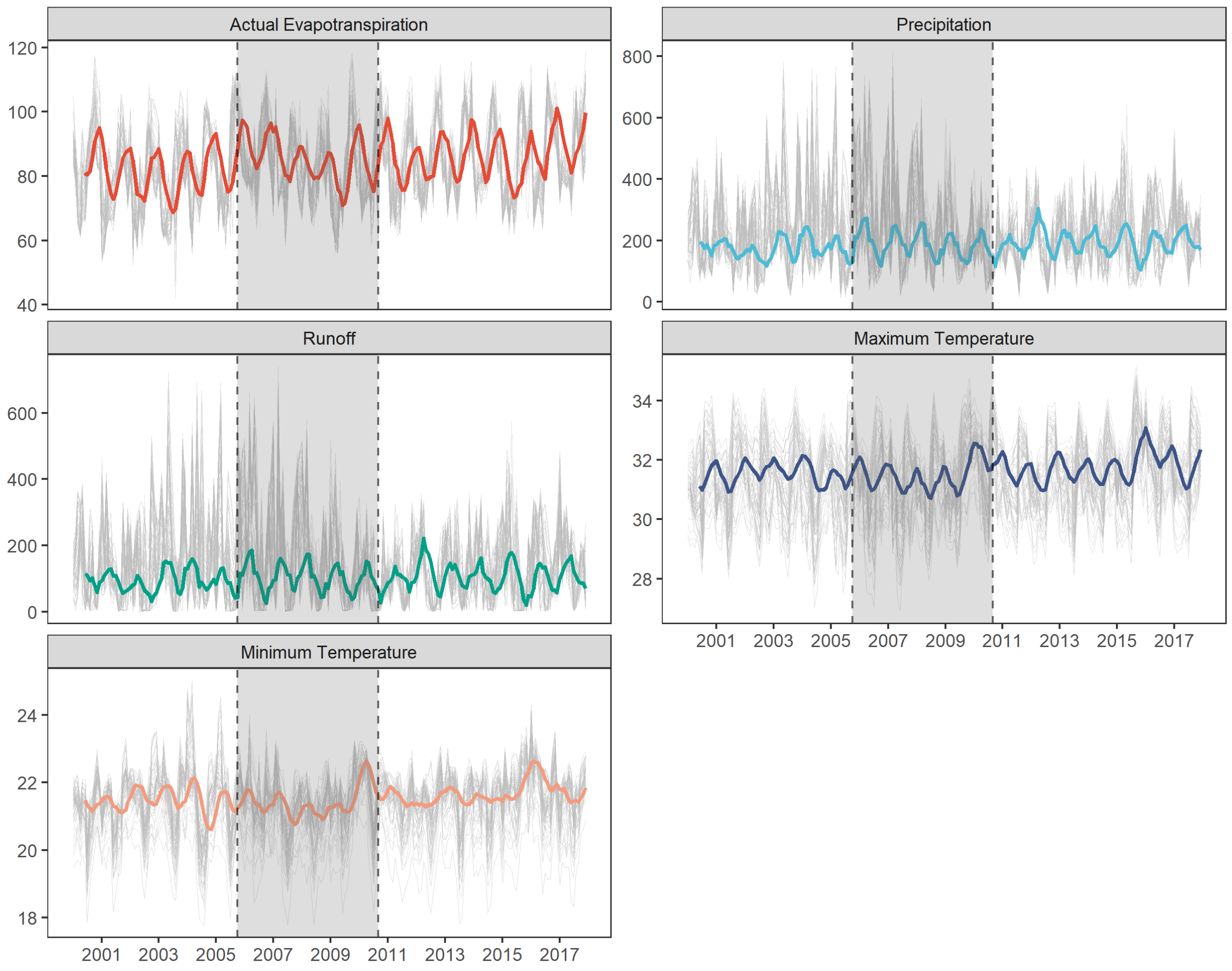

Supplementary Figure 2. 6-month moving averages of the meteorological variables from 2000 to 2017 in the province of Datem Del Marañon. Grey lines: Observed measurements at a district level. Colored lines: 6-month moving averages over the observed measurements at a district level.

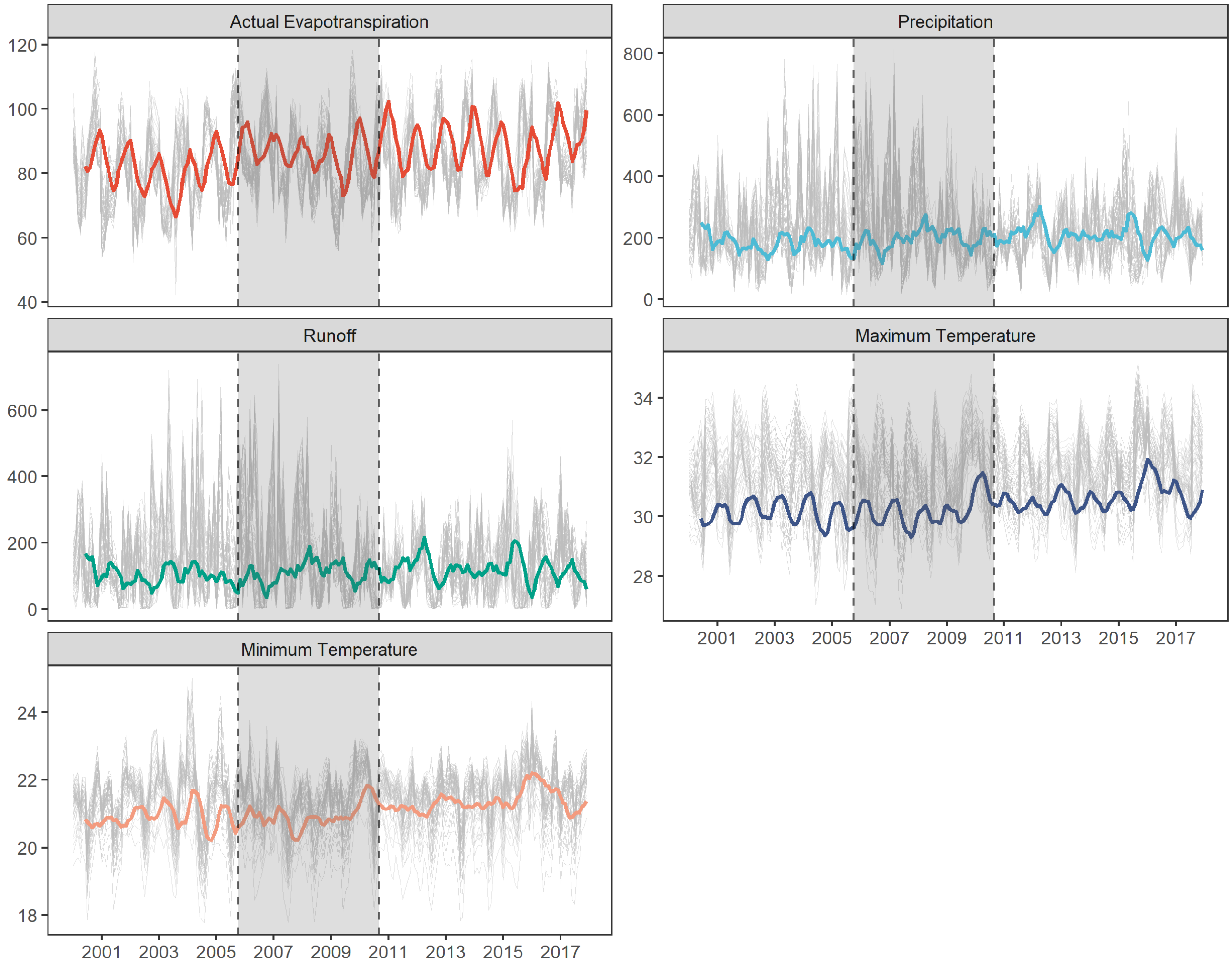

Supplementary Figure 3. 6-month moving averages of the meteorological variables from 2000 to 2017 in the province of Loreto. Grey lines: Observed measurements at a district level. Colored lines: 6-month moving averages over the observed measurements at a district level.

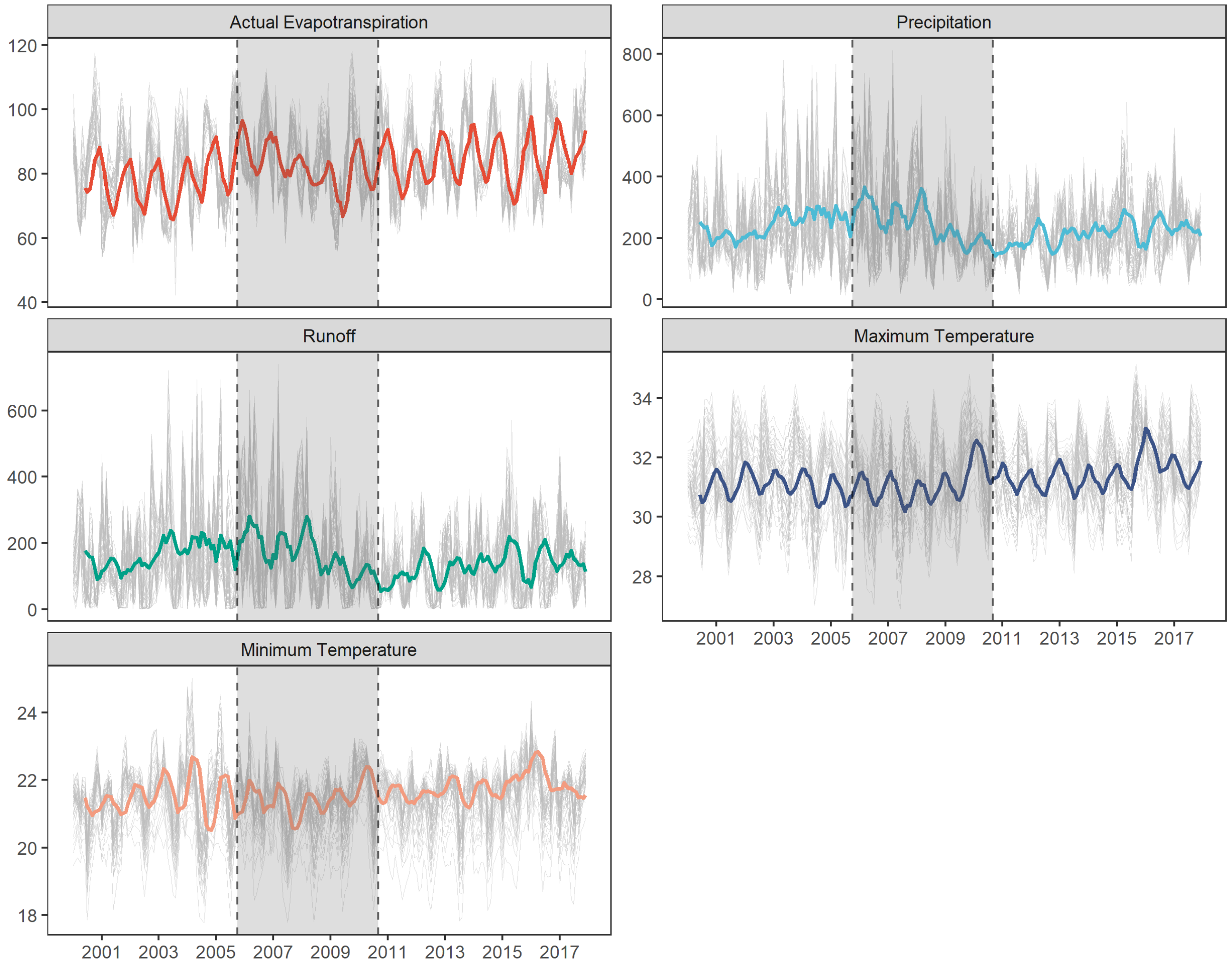

Supplementary Figure 4. 6-month moving averages of the meteorological variables from 2000 to 2017 in the province of Mariscal Ramon Castilla. Grey lines: Observed measurements at a district level. Colored lines: 6-month moving averages over the observed measurements at a district level.

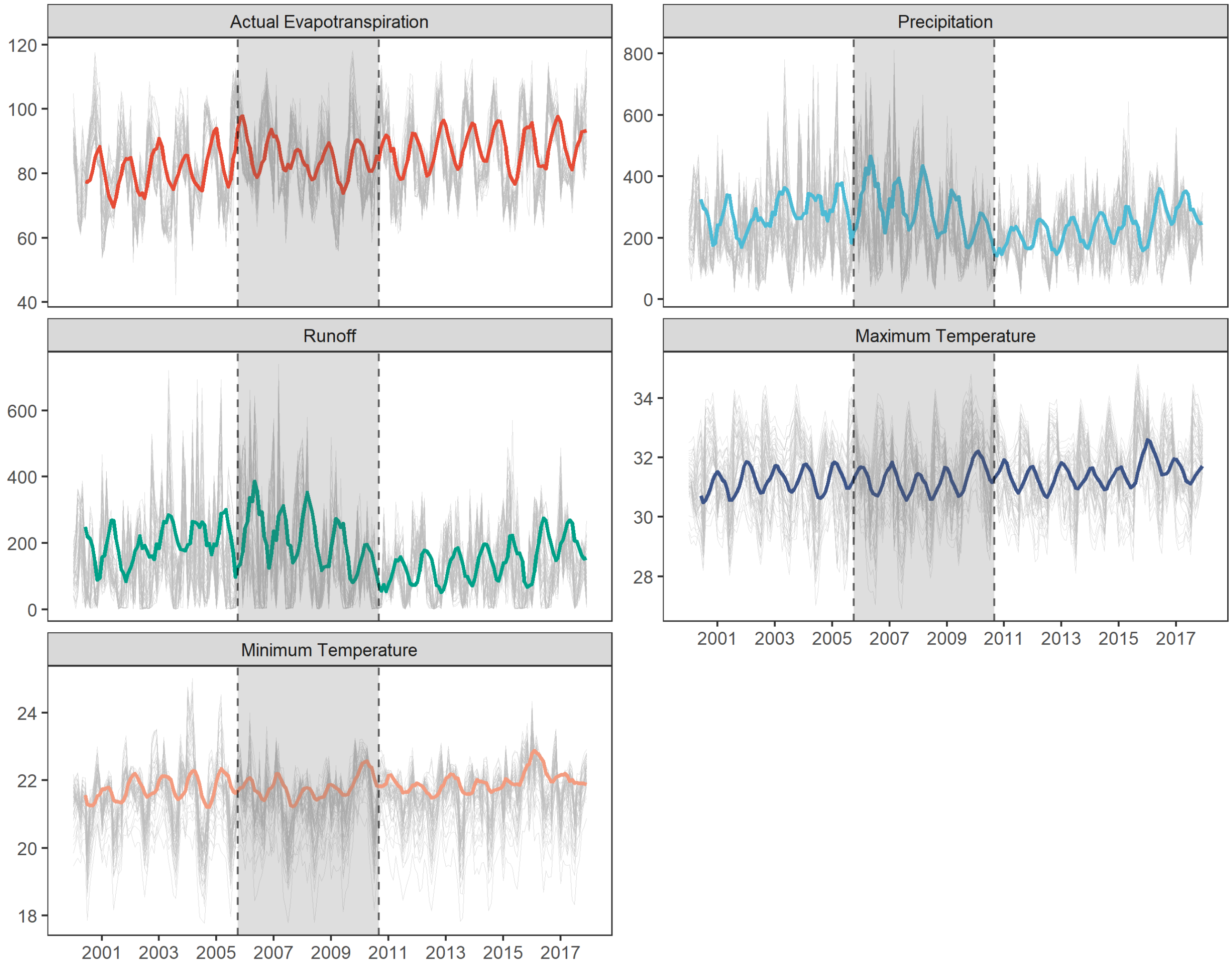

Supplementary Figure 5. 6-month moving averages of the meteorological variables from 2000 to 2017 in the province of Maynas. Grey lines: Observed measurements at a district level. Colored lines: 6-month moving averages over the observed measurements at a district level.

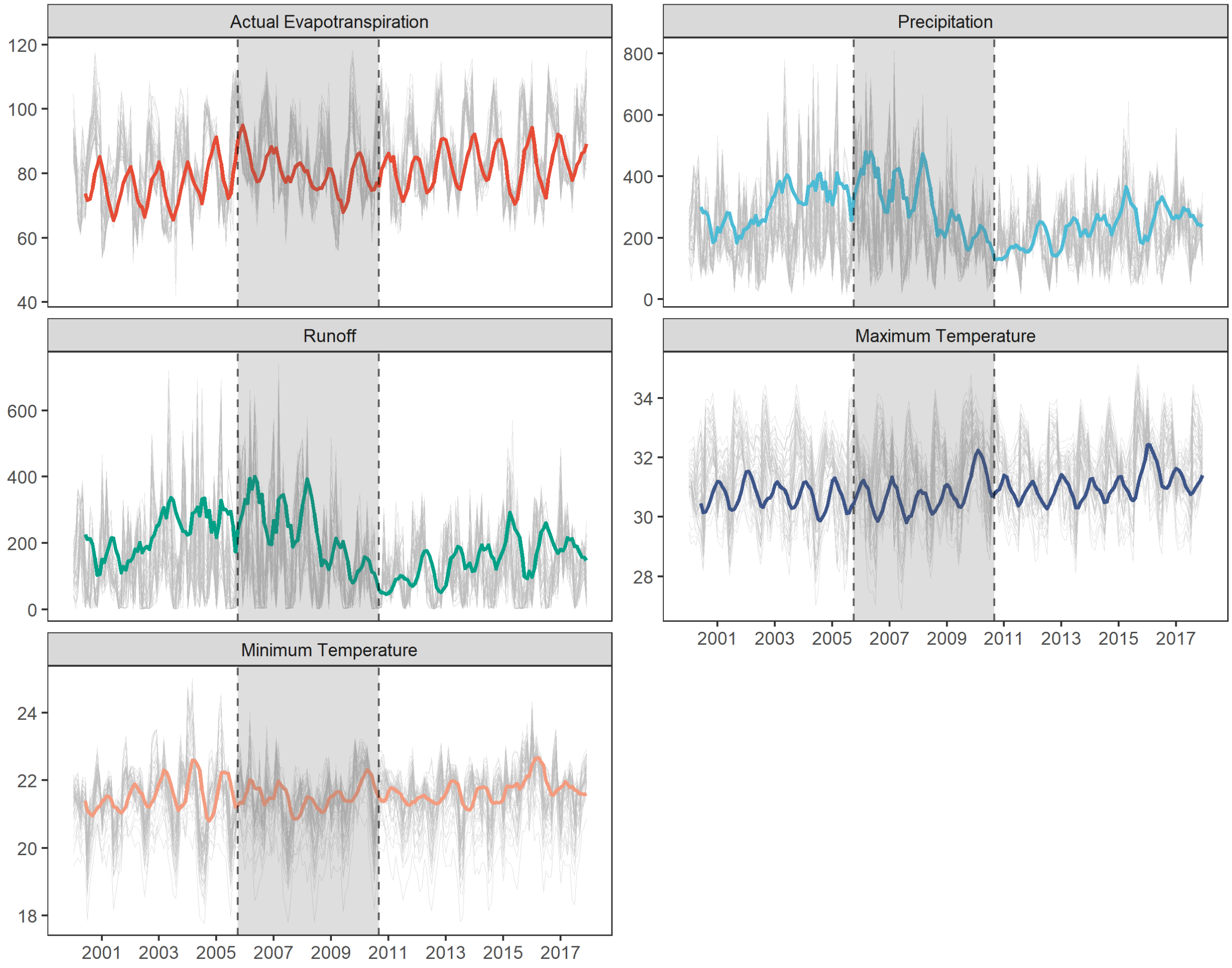

Supplementary Figure 6. 6-month moving averages of the meteorological variables from 2000 to 2017 in the province of Requena. Grey lines: Observed measurements at a district level. Colored lines: 6-month moving averages over the observed measurements at a district level.

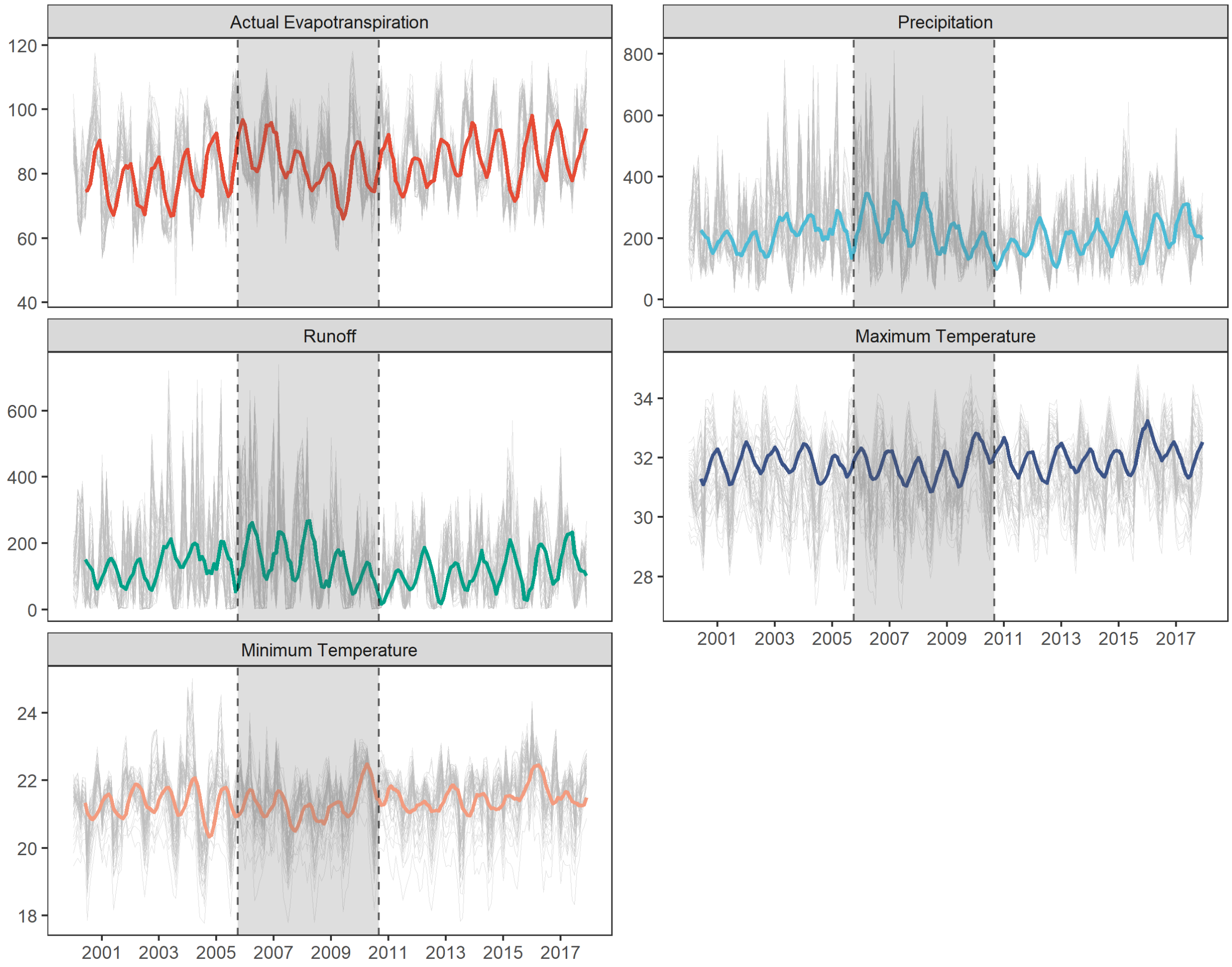

Supplementary Figure 7. 6-month moving averages of the meteorological variables from 2000 to 2017 in the province of Ucayali. Grey lines: Observed measurements at a district level. Colored lines: 6-month moving averages over the observed measurements at a district level.

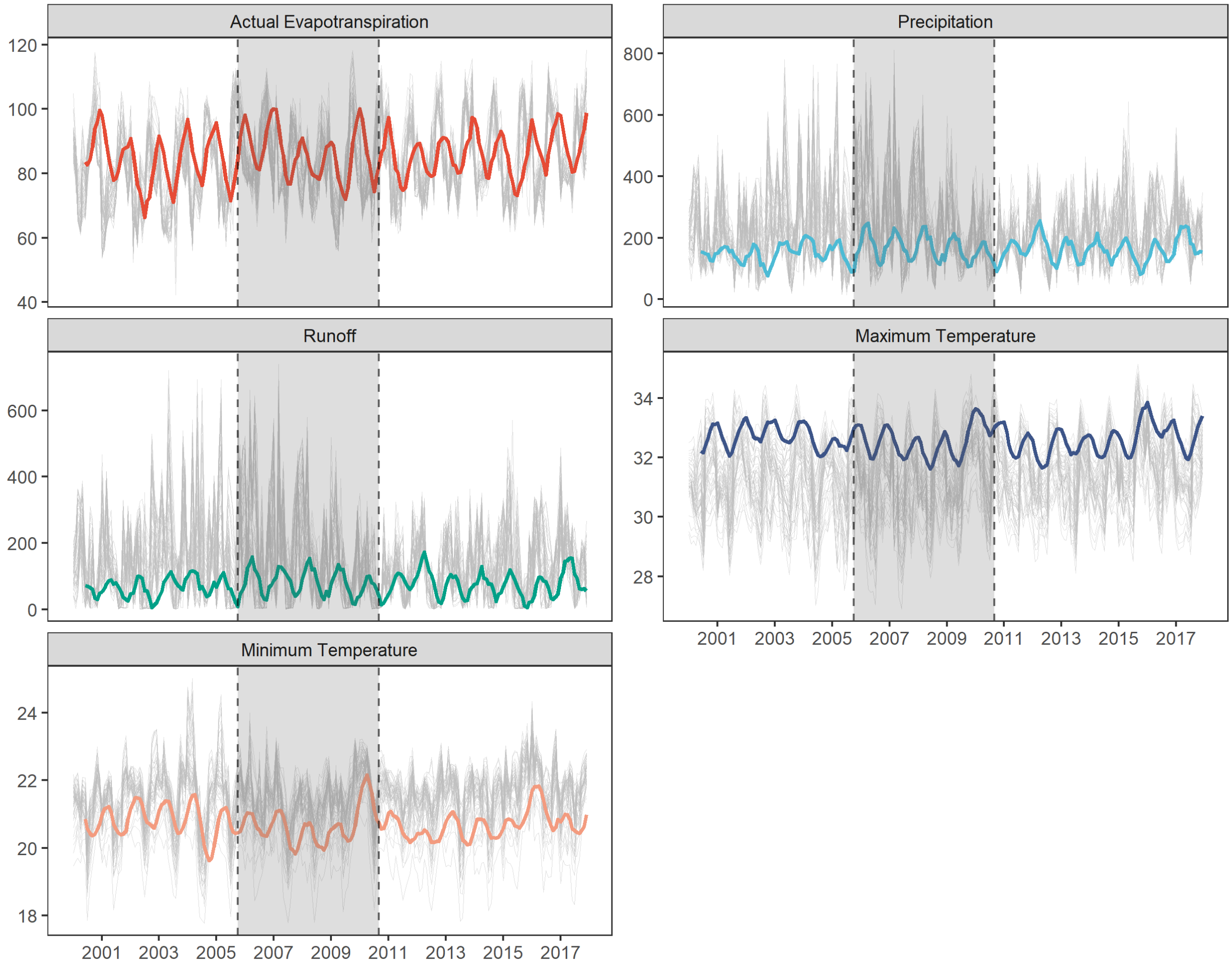

4 Supplementary references

1. Bakdash JZ, Marusich LR. Repeated Measures Correlation. *Front Psychol* (2017) **8**:456. doi:10.3389/fpsyg.2017.00456

2. Hastie T, Tibshirani R. Generalized Additive Models. *Stat Sci* (1986) **1**:297–310.

3. McCullagh P, Nelder JA. *Generalized Linear Models*. 2nd ed. Chapman and Hall/CRC (1989).

4. Hastie T, Tibshirani R. Varying-Coefficient Models. *J R Stat Soc Ser B* (1993) **55**:757–96.

5. Wood SN. Fast stable restricted maximum likelihood and marginal likelihood estimation of semiparametric generalized linear models. *J R Stat Soc Ser B (Statistical Methodol* (2011) **73**:3–36. doi:10.1111/j.1467-9868.2010.00749.x

6. Wood SN. Thin plate regression splines. *J R Stat Soc Ser B (Statistical Methodol* (2003) **65**:95–114. doi:10.1111/1467-9868.00374

7. Wood SN. *Generalized Additive Models: An Introduction with R*. 2nd ed. Chapman and Hall/CRC (2017).
